# SAfety and Feasibility of EArly Resistance Training After Median Sternotomy: The SAFE-ARMS Study

**DOI:** 10.1093/ptj/pzac056

**Published:** 2022-05-13

**Authors:** Jacqueline Pengelly, Stuart Boggett, Adam Bryant, Colin Royse, Alistair Royse, Gavin Williams, Doa El-Ansary

**Affiliations:** Department of Nursing and Allied Health, Swinburne University of Technology, Hawthorn, Victoria, Australia; Department of Exercise & Sport Science, Institute of Health and Wellbeing, Federation University Australia, Mount Helen, Victoria, Australia; Department of Surgery, University of Melbourne, Parkville, Victoria, Australia; Department of Physiotherapy, University of Melbourne, Parkville, Victoria, Australia; Department of Nursing and Allied Health, Swinburne University of Technology, Hawthorn, Victoria, Australia; Department of Surgery, University of Melbourne, Parkville, Victoria, Australia; Department of Anaesthesia and Pain Management, Royal Melbourne Hospital, Parkville, Victoria, Australia; Outcomes Research Consortium, Cleveland Clinic, Cleveland, Ohio, USA; Department of Nursing and Allied Health, Swinburne University of Technology, Hawthorn, Victoria, Australia; Department of Surgery, University of Melbourne, Parkville, Victoria, Australia; Department of Cardiothoracic Surgery, Royal Melbourne Hospital, Parkville, Victoria, Australia; Department of Physiotherapy, University of Melbourne, Parkville, Victoria, Australia; Department of Nursing and Allied Health, Swinburne University of Technology, Hawthorn, Victoria, Australia; Department of Surgery, University of Melbourne, Parkville, Victoria, Australia; Clinical Research Institute, Westmead Private Hospital, Westmead, New South Wales, Australia

**Keywords:** Cardiothoracic Surgery, Exercise, Rehabilitation, Resistance Training, Ultrasound

## Abstract

**Objective:**

The purpose of this study was to determine the safety and feasibility of subacute upper limb resistance exercise on sternal micromotion and pain and the reliability of sternal ultrasound assessment following cardiac surgery via median sternotomy.

**Methods:**

This experimental study used a pretest–posttest design to investigate the effects of upper limb resistance exercise on the sternum in patients following their first cardiac surgery via median sternotomy. Six bilateral upper limb machine-based exercises were commenced at a base resistance of 20 lb (9 kg) and progressed for each participant. Sternal micromotion was assessed using ultrasound at the mid and lower sternum at 2, 8, and 14 weeks postsurgery. Intrarater and interrater reliability was calculated using intraclass correlation coefficients (ICCs). Participant-reported pain was recorded at rest and with each exercise using a visual analogue scale.

**Results:**

Sixteen adults (n = 15 males; 71.3 [SD = 6.2] years of age) consented to participate. Twelve participants completed the study, 2 withdrew prior to the 8-week assessment, and 2 assessments were not completed at 14 weeks due to assessor unavailability. The highest median micromotion at the sternal edges was observed during the bicep curl (median = 1.33 mm; range = −0.8 to 2.0 mm) in the lateral direction and the shoulder pulldown (median = 0.65 mm; range = −0.8 to 1.6 mm) in the anterior–posterior direction. Furthermore, participants reported no increase in pain when performing any of the 6 upper limb exercises. Interrater reliability was moderate to good for both lateral–posterior (ICC = 0.73; 95% CI = 0.58 to 0.83) and anterior–posterior micromotion (ICC = 0.83; 95% CI = 0.73 to 0.89) of the sternal edges.

**Conclusion:**

Bilateral upper limb resistance exercises performed on cam-based machines do not result in sternal micromotion exceeding 2.0 mm or an increase in participant-reported pain.

**Impact:**

Upper limb resistance training commenced as early as 2 weeks following cardiac surgery via median sternotomy and performed within the safe limits of pain and sternal micromotion appears to be safe and may accelerate postoperative recovery rather than muscular deconditioning.

## Introduction

Median sternotomy remains the gold standard surgical incision for optimal access to the heart and vasculature for cardiac surgery.^1–3^ However, patients can experience deficits in physical and functional recovery for several months after surgery, impacting performance of activities of daily living.^4^^,^^5^ A disconnect exists between routine restrictive sternal precautions, such as carrying weights >4.5 kg, and emerging evidence, which poses a challenge for exercise professionals to deliver evidence-based rehabilitation.^6^^,^^7^

Sternal precaution development was founded on the belief that early postoperative (1–6 weeks) arm movements increase the mechanical forces acting in opposition to the holding strength of wire sutures (lateral movement), thus jeopardizing the sternal wiring and skin integrity and resulting in sternal dehiscence.^2–8^ In a study of sternal closure techniques in cadavers, McGregor et al^1^ suggested that activation of the muscles of the anterior chest may result in distractive forces and micromotion of the bone edges >2.0 mm,^1^ which in turn may jeopardize the integrity of the healing sternum. However, a recent study has shown that unweighted unilateral and bilateral upper limb movements, performed within 6 weeks poststernotomy, were not detrimental to bone healing, were well tolerated, decreased postsurgical pain, and resulted in <2.0 mm of sternal micromotion, assessed using the Sternal Instability Scale and sternal ultrasound.^9^ Furthermore, coughing was found to result in the greatest sternal separation during the early postoperative period.^9^ Subsequently, evidence has prompted a paradigm shift towards promotion of upper limb exercise within the same limits of pain and discomfort.^10–12^

A recent systematic review of resistance training following median sternotomy^7^ found that none of the 18 included studies met all American College of Sports Medicine Cardiac Rehabilitation resistance training guidelines in regard to program length (4–6 months), intensity (40%–50% maximal voluntary contraction), frequency (2–3 d/wk), session duration (2–4 sets, 12–15 reps, 8–10 exercises), and resistance (0.5–0.9 kg in the first 12 weeks poststernotomy).^13^ Despite the American College of Sports Medicine recommendation that sternal stability be assessed before commencing upper limb resistance exercise, no studies reported that they assessed sternal stability prior to resistance training commencement or progression.^7^

It is common practice for resistance exercise to be prescribed more conservatively than guidelines recommend, possibly attributable to the lack of evidence investigating the effect of upper limb resistance exercises on sternal healing. Therefore, the aims of this study were to determine whether (1) bilateral machine-based resistance training commenced 2 weeks following cardiac surgery via median sternotomy is safe and feasible, and (2) sternal ultrasound during bilateral resistance training is a reliable measure of sternal micromotion. We hypothesized that (1) the seated row would result in the greatest amount of sternal edge micromotion at 2 weeks postoperatively, and (2) weighted upper limb exercises (eg, triceps dip, bicep curl, shoulder pulldown, shoulder press, lateral raise, and seated row) would not exceed 2.0 mm of sternal edge separation at any time point (2, 8, and 14 weeks postoperatively).

## Methods

### Design

This was a pretest–posttest design study, nested within a pilot randomized controlled trial,^14^ conducted in accordance with the National Statement on Ethical Human Research and the Australian Code for the Responsible Conduct of Research. Ethical approval was granted by the Melbourne Health Human Research Ethics Committee (Application ID: 2017.266). Local governance approval was obtained prior to recruitment commencement. The study followed the CONsolidated Standards of Reporting Trials 2010 guidelines. This trial was prospectively registered with the Australian New Zealand Clinical Trials Registry (ACTRN12617001430325p).

Participants were in the resistance training arm of the Supervised Early Resistance Training study.^14^ The protocol of the primary study, detailing recruitment, inclusion and exclusion criteria, randomization, allocation concealment and blinding, has been described previously.^15^ Written informed participant consent was obtained prior to data collection.

### Role of the Funding Source

The funders played no role in the design, conduct, or reporting of this study.

### Participants

Data from participants in the resistance training group of a pilot randomized controlled trial were analyzed in this study.^14^ Participants were adults undergoing cardiac surgery via median sternotomy at the Royal Melbourne or Melbourne Private hospitals between April 16, 2018, and August 31, 2019. Eligibility criteria for the pilot randomized controlled trial included (1) first elective cardiac surgery via median sternotomy, (2) no diagnosed cognitive impairment, (3) no musculoskeletal conditions limiting exercise ability, (4) sufficient English to provide informed consent and complete surveys, and (5) able to attend the exercise testing site for exercise and/or testing sessions.^14^^,^^15^

### Exercise Intervention

Participants in the resistance training group completed a twice-weekly, moderate-intensity resistance training program for 12 weeks under the supervision of an accredited exercise physiologist. The 6 dynamic upper limb exercises performed on cam-based machines (ie, machines with a cam device that distribute the load evenly throughout the entire exercise range of movement) that were assessed in this study consisted of a seated row, shoulder pulldown, shoulder press, triceps dip, lateral raise, and bicep curl. A single set of each exercise was completed to volitional fatigue (typically 10–15 repetitions). The exercise intervention was previously reported.^14^^,^^15^

Each cam-based resistance machine was adjusted for each participant to ensure optimal comfort and uniformity of the testing set-up and exercise performance (Supplementary Materials A & B).

### Outcome Measures

#### Sternal Micromotion

Real-time sternal images were obtained using a L38v 9 cm linear transducer (10–5 MHz) and Fujifilm SonoSite iViz ultrasound (SonoSite Australasia Pty Ltd, Brookvale, NSW, Australia). Sternal edge micromotion was measured using the annotation and measurement function of the Fujifilm Prosolv5 Synapse Cardiovascular software program (Fujifilm Australia). Measurement of sternal edge separation and/or overlap was recorded in the lateral (coronal plane) and anterior–posterior (sagittal plane) directions in millimeters.^16^

#### Sternal Pain

Sternal pain was evaluated using the Visual Analogue Scale because this has been reported as a valid and reliable measure of postoperative pain.^17^ Participants were required to rate the maximal amount of sternal pain they experienced at rest and during each of the 6 upper limb tasks, with any increase in pain from rest during the 6 upper limb resistance exercises being recorded. Pain was rated on a scale of 0 to 10, with a rating of 0 corresponding to no sternal pain and a rating of 10 indicative of the worst possible pain.^17^ Pain was not recorded during the cough because it was used as a comparator with upper limb resistance exercise sternal micromotion.

### Testing Procedure

Sternal motion testing occurred during 3 testing sessions: (1) at 2 weeks, (2) at 8 weeks, and (3) at 14 weeks postoperatively. To minimize systemic errors of measurement, these sessions were standardized and ultrasound images obtained by a single assessor (J.P.). The assessor received training in sternal ultrasound imaging by a senior physiotherapist, who had developed and validated this measurement technique,^16^ in addition to a cardiothoracic surgeon. The assessor acquired 1 year of experience obtaining sternal ultrasound images in the clinical setting prior to study commencement.

A single, 2-dimensional, 15-second ultrasound video was taken capturing motion at the sternal edges at rest (baseline measurement) during a maximal cough and 6 dynamic upper limb resistance exercises. Sternal ultrasound measurement occurred throughout the entire range of movement for the 6 exercises.

Markings were placed on the participant’s skin with a surgical marker at points 6 cm (mid-sternum) and 10 cm (lower sternum) distal from the sternal notch. The ultrasound transducer was placed directly onto the skin, with minimal pressure applied, throughout the duration of the aforementioned activities. Ultrasound images were obtained at both the mid- and lower-sternum locations for each activity. The activities were repeated if the transducer moved during the movement to obtain an optimal image. Participants were instructed to perform a maximal cough, as hard as possible, and each of the upper limb exercises within their comfortable range of motion to minimize participant burden. Although the order of upper limb exercises was not randomized, the order of performance was dependent on exercise machine availability at the time of the scheduled sessions. The above procedure was repeated at each testing session for each time point reported.

### Ultrasound Image Analysis Procedure

**Figure 1:**
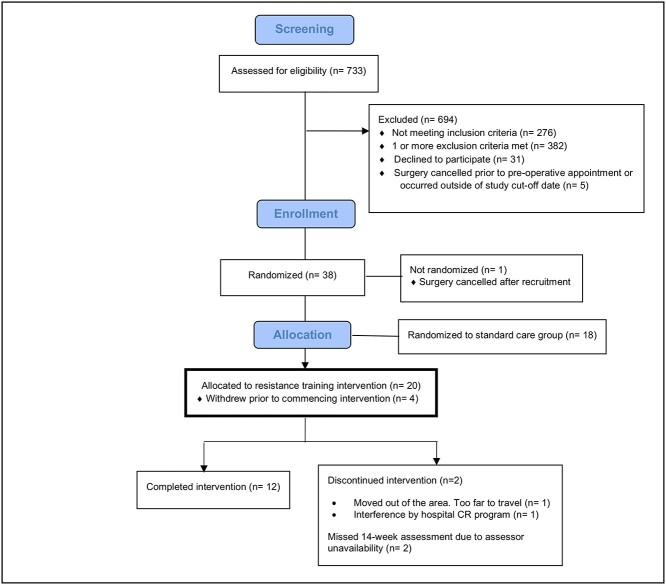
Ultrasound image analysis procedure. (A) Identification of sternal edge tips. (B) Horizontal and vertical axis. (C) Vertical (anterior–posterior) vector measurement. (D) Horizontal (lateral) vector measurement.

Each ultrasound video was viewed in real-time to determine the frame in which (1) the sternal edges could clearly be viewed in the resting position, and (2) the maximum movement for the exercise occurred. Frame-by-frame video analysis was used to determine the point of maximum movement. Straight-line annotations representing the vertical and horizontal axes were drawn through the left and right edges of each hemi-sterna (Fig. 1A and B) to assist with measurement of the vertical and horizontal vectors between the sternal edges. Vertical vector measurement was taken from the right sternal edge tip to the intersection of the left sternal edge intersection (upper and lower points of axes intercepts) (Fig. 1C). Horizontal vector measurement was taken from the tip of the left sternal edge to the intersection of the axes (Fig. 1D). Sternal separation that increased from rest (ie, sternal edges moved further apart) was denoted by a positive value, and sternal separation that decreased from rest (ie, sternal edges moved closer together) was denoted by a negative value.

### Reliability

Eighty ultrasound images were randomly selected using a computer-generated sequence from Research Randomizer (randomizer.org). To evaluate intrarater and interrater reliability, 2 raters independently assessed each of the 80 images twice, with 1 month between each assessment. Rater 1 (J.P.) was deemed to have intermediate experience (>100 hours) completing sternal ultrasound measurement using Prosolv5, whereas Rater 2 (S.B.) was deemed a novice (<20 hours experience). To ensure Rater 2 was familiar with the study protocol, including identification of the sternal edges, image annotation, and the methodology of obtaining measurements, Rater 2 attended four 1-hour training sessions under the guidance of Rater 1. Three fidelity checks of 10 ultrasound images were also conducted as part of the training and analysis process, which were cross-checked with Rater 1.

**Figure 2:**
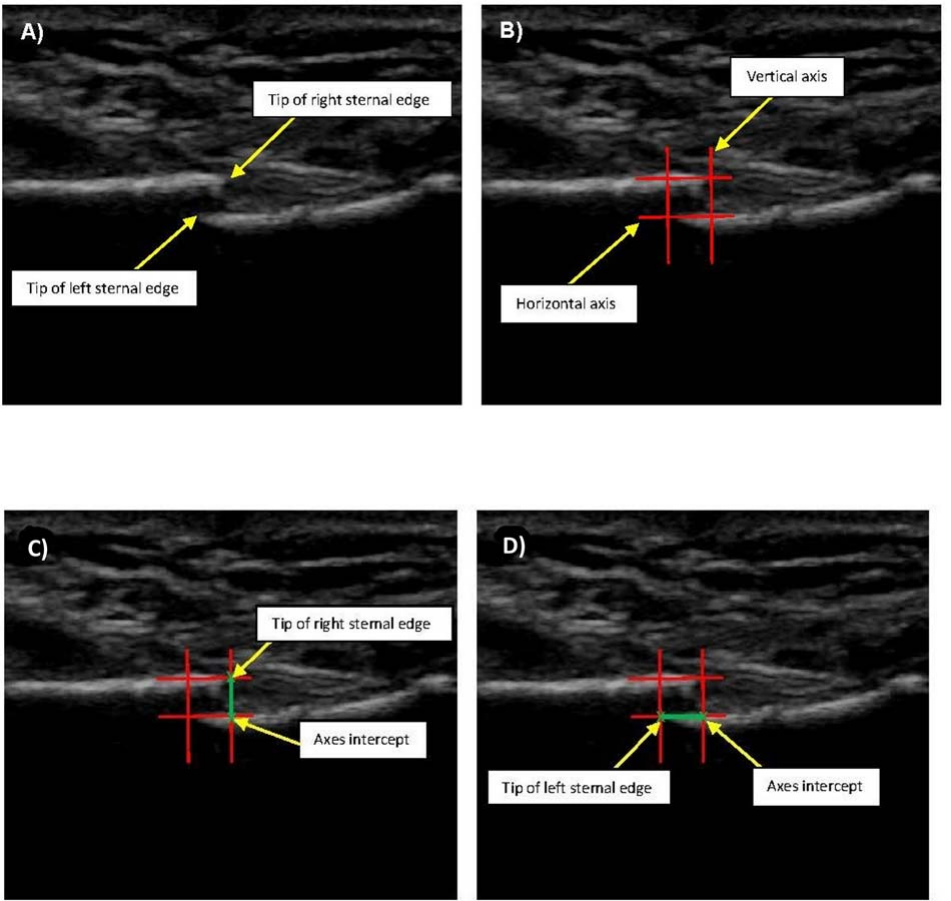
Summary of participant screening and recruitment.

### Statistical Analysis

Continuous participant demographic data (age) were reported as mean and SD, whereas categorical participant demographic data (sex assigned at birth, comorbidities, surgical procedure, and sternal closure method) were reported as the absolute number of participants and percentage of the study population. Postoperative days to start of intervention was reported as median and interquartile range values. Safety during each exercise was determined using lateral and anterior–posterior sternal edge micromotion values measured by the more experienced assessor (J.P.) and was compared across the 3 time points, reported as median and minimum and maximum values. Intraclass correlation coefficient (ICC) estimates and their 95% CIs were used to calculate intrarater and interrater reliability. Intrarater reliability was calculated for each of the 2 raters individually, based on a consistency, 2-way mixed-effects model. To determine the reliability of measurements between raters, ICC interrater reliability was calculated based on a mean-rating (k = 2), absolute-agreement, 2-way mixed-effects model. Reliability was categorized according to Koo and Li^18^ ICC value classifications (<0.50 = poor, 0.50–0.75 = moderate, 0.75–0.90 = good, >0.90 = excellent). All statistical analyses were undertaken using IBM SPSS Statistics version 26 (IBM Corporation, Armonk, New York, USA).

## Results

### Participant Demographics

Of the 20 participants randomized to the resistance training group, 4 withdrew following hospital discharge but prior to intervention commencement. A further 2 participants withdrew from the study: 1 relocated out of the area and the other transferred to an alternate cardiac rehabilitation program. A summary of participant screening and enrolment is shown in Figure 2. Additionally, data were not collected for 2 participants at the 14-week assessment due to assessor unavailability, leaving 12 participants with complete data.

The mean age of participants was 71.3 (SD = 6.2) years and comprised of 15 (94%) males. Twelve patients (75%) underwent coronary artery bypass grafting and 4 (25%) had a coronary artery bypass plus a valve repair/replacement.

Median sternotomy closure was achieved via sternal plating (n = 6), conventional stainless-steel wires (n = 6), and stainless-steel cables (n = 4), with the sternal closure method varying according to surgeon preference. The resistance training intervention commenced 14.5 days (interquartile range = 2.5 days) postoperatively. Patients were not taking any prescribed pain medications at the time of commencing the intervention. A summary of baseline patient demographic data is described in Table 1.

**Table 1:** Summary of Patient Demographics at Baseline*^a^*

**Baseline Data**	**Values**
Age, y	71.3 (SD = 6.2)
Sex assigned at birth	
Male	15 (94%)
Female	1 (6%)
Comorbidities	
Overweight	6 (37.5%)
Obese	8 (50%)
Smoking history	10 (62.5%)
Ex-smoker	9 (56%)
Current smoker	1 (6%)
Type 2 diabetes mellitus	8 (50%)
Kidney disease	4 (25%)
Atrial fibrillation	2 (12.5%)
Asthma	2 (12.5%)
Macular degeneration	2 (12.5%)
Surgical procedure	
Coronary artery bypass graft	12 (75%)
CABG + valve	4 (25%)
Sternal closure method	
Plating	6 (37.5%)
Cables	4 (25%)
Wires	6 (37.5%)
Time to intervention commencement	
Postoperative days	14.5 (IQR = 2.5)

*^a^* IQR = interquartile range.

### Reliability

The ICC for interrater reliability for lateral micromotion was between moderate and good (0.73 [0.58–0.83]), whereas intrarater reliability was between good and excellent for rater 1 (0.90 [0.85–0.93]) and between poor and good for rater 2 (0.63 [0.48–0.74]; Tab. 2).

**Figure 3:**
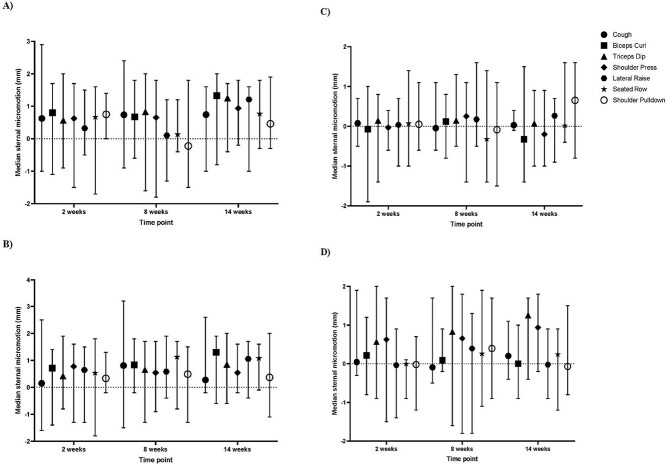
(A) Changes in lateral motion at the sternal edges (mm) during cough, bicep curl, triceps dip, shoulder press, lateral raise, seated row, and shoulder pulldown at the mid-sternum at 2, 8, and 14 weeks postoperatively. (B) Changes in lateral motion at the sternal edges (mm) during cough, bicep curl, triceps dip, shoulder press, lateral raise, seated row, and shoulder pulldown at the lower sternum at 2, 8, and 14 weeks postoperatively. (C) Changes in anterior–posterior motion at the sternal edges (mm) during cough, bicep curl, triceps dip, shoulder press, lateral raise, seated row, and shoulder pulldown at the mid-sternum at 2, 8, and 14 weeks postoperatively. (D) Changes in anterior–posterior motion at the sternal edges (mm) during cough, bicep curl, triceps dip, shoulder press, lateral raise, seated row, and shoulder pulldown at the lower sternum at 2, 8, and 14 weeks postoperatively.

**Table 2:** ICCs for Intrarater and Interrater Reliability*^a^*

**Intrarater Reliability**	**ICC**	**95% CI**	**Cronbach Alpha**	**Standard Error of Measurement**
Lateral micromotion				
Rater 1	0.90	0.85 to 0.93	0.95	0.46
Rater 2	0.63	0.48 to 0.74	0.77	0.87
Interrater reliability	0.73	0.58 to 0.83	0.74	0.66
Anterior–posterior micromotion				
Rater 1	0.74	0.62 to 0.82	0.85	0.51
Rater 2	0.58	0.41 to 0.71	0.73	0.60
Interrater reliability	0.83	0.73 to 0.89	0.83	0.37

*^a^* ICC = intraclass correlation coefficient.

For anterior–posterior micromotion, ICC interrater reliability was between moderate and good (0.83 [0.73–0.89]). Intrarater reliability was between moderate and good for rater 1 (0.74 [0.62–0.82]) and between poor and moderate for rater 2 (0.58 [0.41–0.71]; Tab. 2).

### Sternal Edge Micromotion

#### Lateral Motion at the Sternal Edges

Median lateral micromotion was highest during the bicep curl (median = 1.33 mm; −0.8 to 2.0 mm) at the mid-sternum at 14 postoperative weeks (Tab. 3; Fig. 3A), whereas the greatest range in lateral movement (4.7 mm) also occurred at the mid-sternum during a cough (median = 0.81 mm; −1.5 to 3.2 mm) at 8 weeks. The lateral raise resulted in the lowest median movement at 0.10 mm (−1.3 to 1.2 mm) at the lower sternum at 8 weeks (Tab. 3; Fig. 3B). No patients exceeded the 2-mm micromotion threshold for any upper limb exercise performed.

**Table 3:** Median (Minimum to Maximum) Sternal Micromotion (mm) for Each Activity at 2, 8, and 14 Week Postsurgery*^a^*

Activity	2 Weeks (n = 16)		8 Weeks (n = 14)		14 Weeks (n = 12)	
	Micromotion	Pain	Micromotion	Pain	Micromotion	Pain
Lateral mid-sternum						
Cough	0.15 (−1.60 to 2.50)	N/A	0.81 (−1.5 to 3.2)	N/A	0.28 (−0.2 to 2.6)	N/A
Bicep curl	0.72 (−1.4 to 1.4)	0/10 (0)	0.83 (1.5 to 3.2)	0/10 (0)	1.30 (−0.6 to 1.9)	0/10 (0)
Triceps dip	0.42 (−0.8- to 1.9)	0/10 (0)	0.65 (−1.3 to 1.7)	0/10 (0)	0.85 (−0.6 to 2.0)	0/10 (0)
Shoulder press	0.77 (−1.3 to 1.6)	0/10 (0)	0.54 (−0.9 to 1.7)	0/10 (0)	0.55 (−0.2 to 1.6)	0/10 (0)
Lateral raise	0.64 (−1.3 to 1.5)	0/10 (0)	0.58 (0.4 to 1.9)	0/10 (0)	1.05 (−0.4 to 1.7)	0/10 (0)
Seated row	0.54 (−1.8 to 1.8)	0/10 (0)	1.13 (−0.8 to 1.7)	0/10 (0)	1.08 (−0.1 to 1.6)	0/10 (0)
Shoulder pulldown	0.33 (−0.2 to 1.3)	0/10 (0)	0.49 (−1.3 to 1.5)	0/10 (0)	0.37 (−1.1 to 2.0)	0/10 (0)
Lateral lower sternum						
Cough	0.63 (−1.0 to 2.9)	N/A	0.74 (−0.9 to 2.4)	N/A	0.74 (−1.0 to 1.6)	N/A
Bicep curl	0.80 (−1.1 to 1.7)	0/10 (0)	0.68 (−0.6 to 1.8)	0/10 (0)	1.33 (−0.8 to 2.0)	0/10 (0)
Triceps dip	0.57 (−0.9 to 2.0)	0/10 (0)	0.83 (−1.6 to 2.0)	0/10 (0)	1.26 (−0.4 to 1.7)	0/10 (0)
Shoulder press	0.63 (−1.5 to 1.7)	0/10 (0)	0.65 (−1.8 to 1.8)	0/10 (0)	0.94 (−1.3 to 1.3)	0/10 (0)
Lateral raise	0.32 (−0.5 to 1.5)	0/10 (0)	0.10 (−1.3 to 1.2)	0/10 (0)	1.21 (−1.0 to 1.6)	0/10 (0)
Seated row	0.67 (−1.7 to 1.6)	0/10 (0)	0.14 (−0.4 to 1.2)	0/10 (0)	0.77 (−0.3 to 1.8)	0/10 (0)
Shoulder pulldown	0.75 (0.0 to 1.4)	0/10 (0)	−0.22 (−1.5 to 1.8)	0/10 (0)	0.46 (−0.3 to 1.9)	0/10 (0)
Anterior–posterior mid-sternum						
Cough	0.08 (−0.5 to 0.7)	N/A	−0.05 (−0.6 to 1.1)	N/A	0.03 (−0.1 to 0.4)	N/A
Bicep curl	−0.07 (−1.9 to 1.0)	0/10 (0)	0.12 (−0.8 to 0.8)	0/10 (0)	−0.33 (−1.4 to 1.5)	0/10 (0)
Triceps dip	0.15 (−1.4 to 0.8)	0/10 (0)	0.15 (−0.5 to 1.3)	0/10 (0)	0.07 (−1.0 to 0.9)	0/10 (0)
Shoulder press	−0.04 (−0.6 to 0.4)	0/10 (0)	0.25 (−1.4 to 1.1)	0/10 (0)	−0.20 (−1.0 to .9)	0/10 (0)
Lateral raise	0.04 (−1.0 to 0.7)	0/10 (0)	0.18 (−0.5 to 1.6)	0/10 (0)	0.27 (−0.9 to 0.7)	0/10 (0)
Seated row	0.07 (−1.0 to 1.4)	0/10 (0)	−0.32 (−1.4 to 1.4)	0/10 (0)	0.02 (−0.4 to 1.6)	0/10 (0)
Shoulder pulldown	0.05 (−0.6 to 1.1)	0/10 (0)	−0.09 (−1.5 to 1.1)	0/10 (0)	0.65 (−0.8 to 1.6)	0/10 (0)
Anterior–posterior lower sternum						
Cough	0.04 (−0.3 to 1.9)	N/A	−0.09 (−0.5 to 1.7)	N/A	0.20 (−0.4 to 1.1)	N/A
Bicep curl	0.22 (−0.8 to 1.2)	0/10 (0)	0.09 (−0.2 to 0.9)	0/10 (0)	0.00 (−0.9 to 1.0)	0/10 (0)
Triceps dip	0.08 (−0.9 to 1.0)	0/10 (0)	0.09 (−0.6 to 1.0)	0/10 (0)	−0.02 (−1.3 to 1.3)	0/10 (0)
Shoulder press	0.20 (−1.0- to 1.3)	0/10 (0)	−0.08 (−0.6 to 0.9)	0/10 (0)	0.15 (−0.6 to 0.8)	0/10 (0)
Lateral raise	−0.04 (−1.4 to 0.9)	0/10 (0)	0.39 (−1.8 to 1.3)	0/10 (0)	−0.02 (−0.9 to 0.9)	0/10 (0)
Seated row	0.00 (−0.9 to 0.1)	0/10 (0)	0.26 (−1.1 to 1.9)	0/10 (0)	0.24 (−1.2 to 0.9)	0/10 (0)
Shoulder pulldown	−0.02 (−1.2 to 0.7)	0/10 (0)	0.40 (−0.9 to 1.7)	0/10 (0)	−0.07 (−0.8 to 1.5)	0/10 (0)

*^a^* N/A = not assessed.

#### Anterior–Posterior Motion at the Sternal Edges

Anterior–posterior micromotion is depicted in Figure 3C for the mid-sternum and Figure 3D for the lower sternum. The greatest sternal movement was a 0.65-mm (−0.8 to 1.6 mm) increase in separation from rest at the mid-sternum, which occurred during the shoulder pulldown at 14 postoperative weeks (Tab. 3; Fig. 3C). The highest variability in movement was 3.1 mm, which occurred at the lower sternum during the lateral raise at 8 weeks (median = 0.39 mm; −1.8 to 1.3 mm; Tab. 3; Fig. 3D). The lowest median anterior–posterior movement occurred at the lower sternum during the seated row at 2 weeks (median = 0.00 mm; −0.9 to 1.0 mm) and the bicep curl at 14 weeks (median = 0.00 mm; −0.9 to 1.0 mm; Tab. 3; Fig. 3D). No patients exceeded the 2-mm micromotion threshold for any upper limb exercise performed.

Secondary analysis of sternal micromotion for each exercise according to the sternal closure mechanism was also undertaken and is reported in Supplementary Material C, Supplementary Table 1, and Supplementary Figures 1, 2, 3, and 4.

### Sternal Pain

There was no increase in participant-reported sternal pain, from 0 at rest, during the performance of any of the weighted upper limb exercises (Tab. 3).

### Upper Limb Exercise Resistance and Repetitions

At 2 weeks postoperatively, all participants completed the 6 upper limb exercises using a 20-lb weight (the lowest machine resistance) for 10 repetitions (Supplementary Appendix 1). At the subsequent time points, the greatest increase in mean weight lifted occurred with the triceps dip at 8 weeks and the shoulder pulldown at 14 weeks, with patients completing 12 repetitions at an average of 81.1 lb (SD = 26.9 lb) and 90.9 lb (SD = 50.4), respectively (Supplementary Appendix 1).

## Discussion

This study found that bilateral weighted upper limb exercises performed on cam-based machines that moved in a single plane, in a controlled environment and supervised by an exercise physiologist, are safe. To our knowledge, this study is the first to examine the effects of upper limb resistance exercises on sternal micromotion in the early postoperative period using resistance loads exceeding those required to perform many activities of daily living.^19^^,^^20^ All exercises resulted in ≤2.0-mm sternal micromotion in the lateral and coronal planes at every time point. Sternal healing and osteosynthesis are enhanced by early bone stability.^21^ It has been hypothesized that >2.0 mm of displacement at bony edges of a fracture site may lead to necrosis and impair healing.^1^^,^^21^ McGregor et al^1^ applied lateral, anterior–posterior, rostral-caudal, and simulated Valsalva distractive forces to cadavers that were closed using varied sternal wiring techniques and reported that fractures and complete dehiscence of the sternum may occur when exercise activates the muscles of the anterior chest and displacement of the bone edges is >2.0 mm.^1^

Furthermore, there were no clinical signs and symptoms of sternal complications, such as a significant increase in pain from that reported at rest, clicking or crepitus, or palpable increase in sternal separation or motion during the performance of the 6 upper limb resistance exercises. This finding is consistent with prior research that has reported the safety and efficacy of active unweighted upper limb and trunk tasks in cardiac surgery patient cohorts with uncomplicated sternotomy and sternal instability.^9^^,^^22^ Collectively, this evidence supports an active participatory model of cardiac rehabilitation that engages patients in early active and resisted exercise to reduce pain, facilitate activities of daily living, and optimize recovery, rather than a restrictive postoperative protocol, historically derived from early cadaver studies.^2^^,^^16^^,^^22^

Previous literature pertaining to exercise after cardiac surgery was based on the premise that exercises involving the pectoral muscles act in direct opposition to the wire fixation^8^ and the observation that distraction of the skin was caused by bilateral end-range shoulder extension.^23^ The seated row primarily involves horizontal abduction, extension, and retraction of the shoulder girdle; thus, we hypothesized that the moderate-resistance seated row exercise would result in the greatest amount of sternal micromotion at 2 weeks postoperatively. However, this study found that there was negligible (≤2.0 mm) sternal edge movement at any time point, so no reduction in sternal micromotion was observed over time.

This study concurred with our hypothesis that weighted upper limb exercises would result in sternal micromotion measures <2 mm at every time point recorded. This was independent of the mode of sternal closure. Although none of the 6 upper limb resistance exercises exceeded 2.0 mm, performance of a cough did at the mid- and lower sternum in the first 8 weeks postoperatively for 3 participants.

The difference in intrarater and interrater ICC reliability, between raters 1 and 2, indicates that when sternal ultrasound is used to assess sternal edge micromotion, greater experience with obtaining and measuring ultrasound images may increase the reliability of ultrasound measurement. However, it should be noted that in this study we calculated our measures of sternal separation and micromotion on an external platform to ensure a consistent methodology and to assess reliability of calculating the measures. This is not usual practice because most sonographers obtain measures in real-time at the time of image acquisition by using the ultrasound machine calipers.

A strength of the study is that the exercise equipment utilized for resistance training was purpose built to facilitate activation of desired muscle groups and stabilize other body segments by way of seatbelts. This ensured optimal body biomechanics during each exercise without accessory movements and assisted injury risk reduction. The findings also suggest that performing bilateral daily tasks, such as pulling a chair out, opening doors, lifting groceries, or carrying a load of washing,^19^^,^^20^ could be performed safely where patient education on safe lifting techniques is provided.

###  

#### Limitations

One limitation of the study is skin motion with respect to the transducer during performance of the 6 upper limb exercises, which may have contributed to error of measurement. Within this study there was the potential for bias, because both ultrasound assessors did not have the same degree of experience analyzing ultrasound images, which may have resulted in an under-estimation of the ICC reliability. To ensure reliability of the time point comparisons, safety was determined using data from the more experienced assessor.

Another limitation of this pilot study is that the small sample size that completed the testing (n = 12) were predominantly men (n = 11). There are potential differences in bone healing between men and women and particularly in those of advanced age (ie, 60s vs 80s). As such, a larger sample size is warranted to investigate the prevalence of pain or other sternal complications.

Although the findings of this study inform safety and feasibility of early postoperative resistance exercise training, it should be noted that the exercises were performed bilaterally on specialized exercise machines. Thus, the findings may not translate to unilateral upper limb resistance exercises or to home-based equipment or exercises. Because the exercise equipment is not routinely used in broader cardiac rehabilitation programs, it is important that future research investigates the impact of other modes of resistance training (eg, TheraBand and free weights) that can be used within community and residential settings to provide options for exercise intervention and inform cardiac rehabilitation guidelines.

This study found that cam machine-based upper limb resistance exercises, performed as early as 2 weeks following median sternotomy, in the absence of pain and discomfort are safe and feasible following cardiac surgery. Performance of 6 upper limb resistance exercises resulted in sternal micromotion <2.0 mm, which is within the safe limits for bone healing. Future research investigating sternal micromotion during upper limb exercises performed with varying modes of resistance training equipment in a larger cohort is warranted.

## Supplementary Material

Supplementary_Fig_1_pzac056

Supplementary_Fig_2_pzac056

Supplementary_Fig_3_pzac056

Supplementary_Fig_4_pzac056

Supplementary_Table_1_pzac056

Supplement_A_pzac056

Supplement_B_pzac056

Supplement_C_pzac056

## Clinical Trial Registration

This trial was prospectively registered with the Australian New Zealand Clinical Trials Registry (ANZCTR) (ref. no.: ACTRN12617001430325p; universal trial no. [UTN] U1111-1203-2131).

The full protocol in published in Trials (https://doi.org/10.1186/s13063-020-04558-x).

Data from this study were presented at:

(1) The Australian Cardiovascular Health and Rehabilitation Association (ACRA) Virtual Annual Scientific Meeting; August 11, 2020.
(2) The American Association of Cardiovascular and Pulmonary Rehabilitation (AACVPR) Annual Scientific Meeting; September 30, 2020; virtual.
(3) The Cardiac Society of Australia and New Zealand (CSANZ) Annual Scientific Meeting; December 8, 2020; virtual.
(4) Research to Practice 2021, Exercise and Sports Science Australia; May 7, 2021; virtual.
