# Supplementary figures and images for: SAfety and Feasibility of EArly Resistance Training After Median Sternotomy: The SAFE-ARMS Study
Source: Phys Ther. 2022 May 13;102(7):pzac056. doi: 10.1093/ptj/pzac056 (PMC9351378; doi:10.1093/ptj/pzac056)

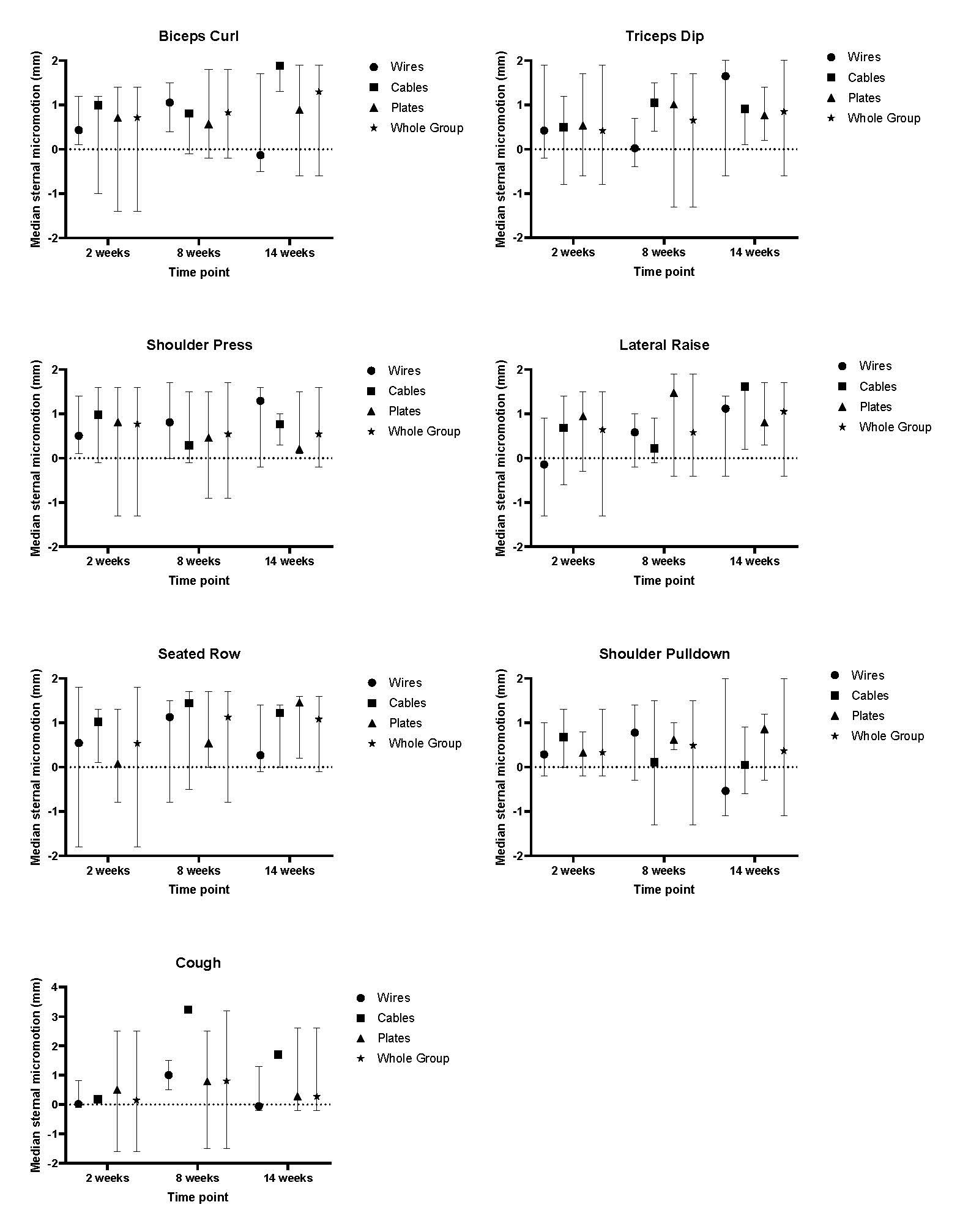

Supplement: Supplementary_Fig_1_pzac056 [file supplementary_fig_1_pzac056.jpeg]

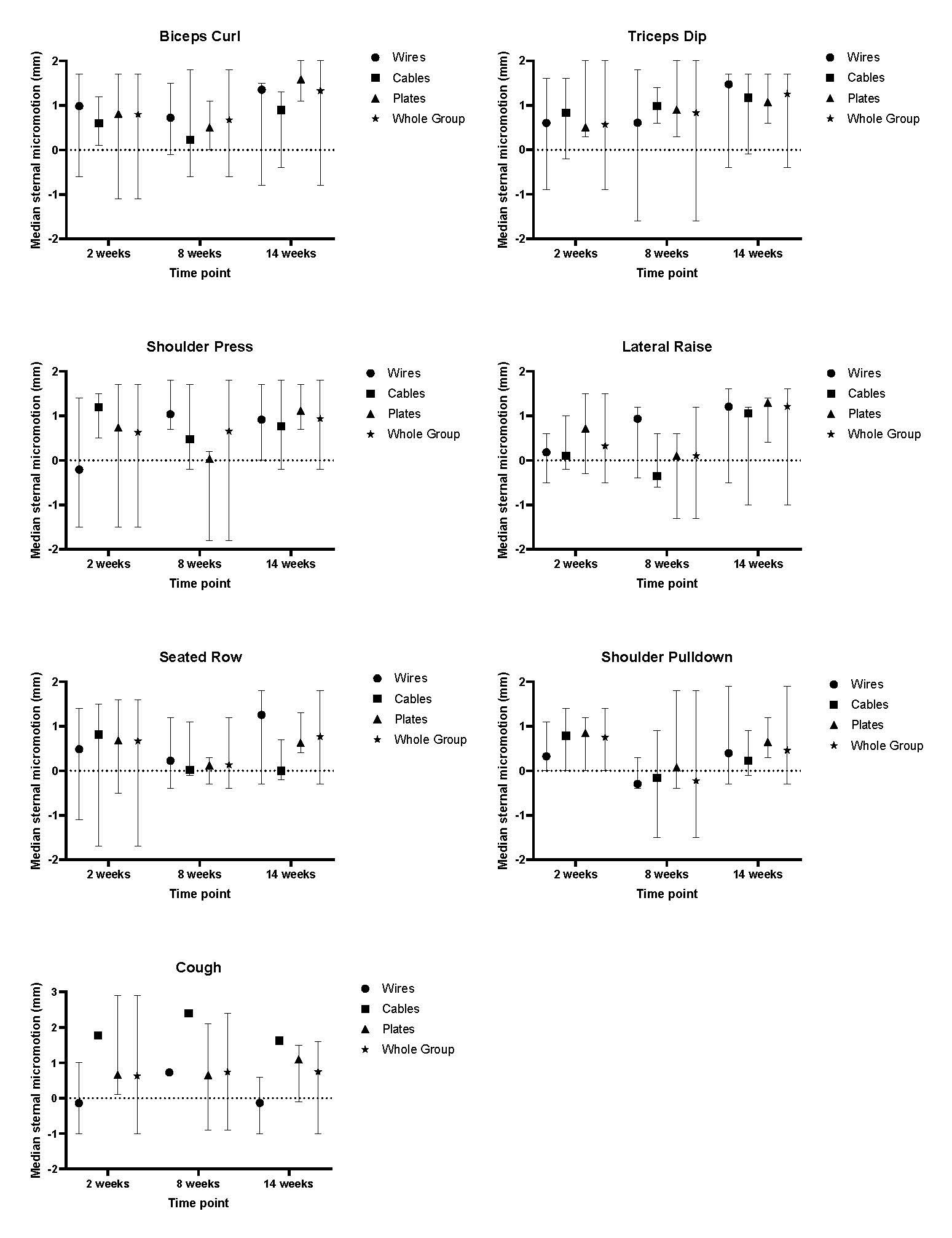

Supplement: Supplementary_Fig_2_pzac056 [file supplementary_fig_2_pzac056.jpeg]

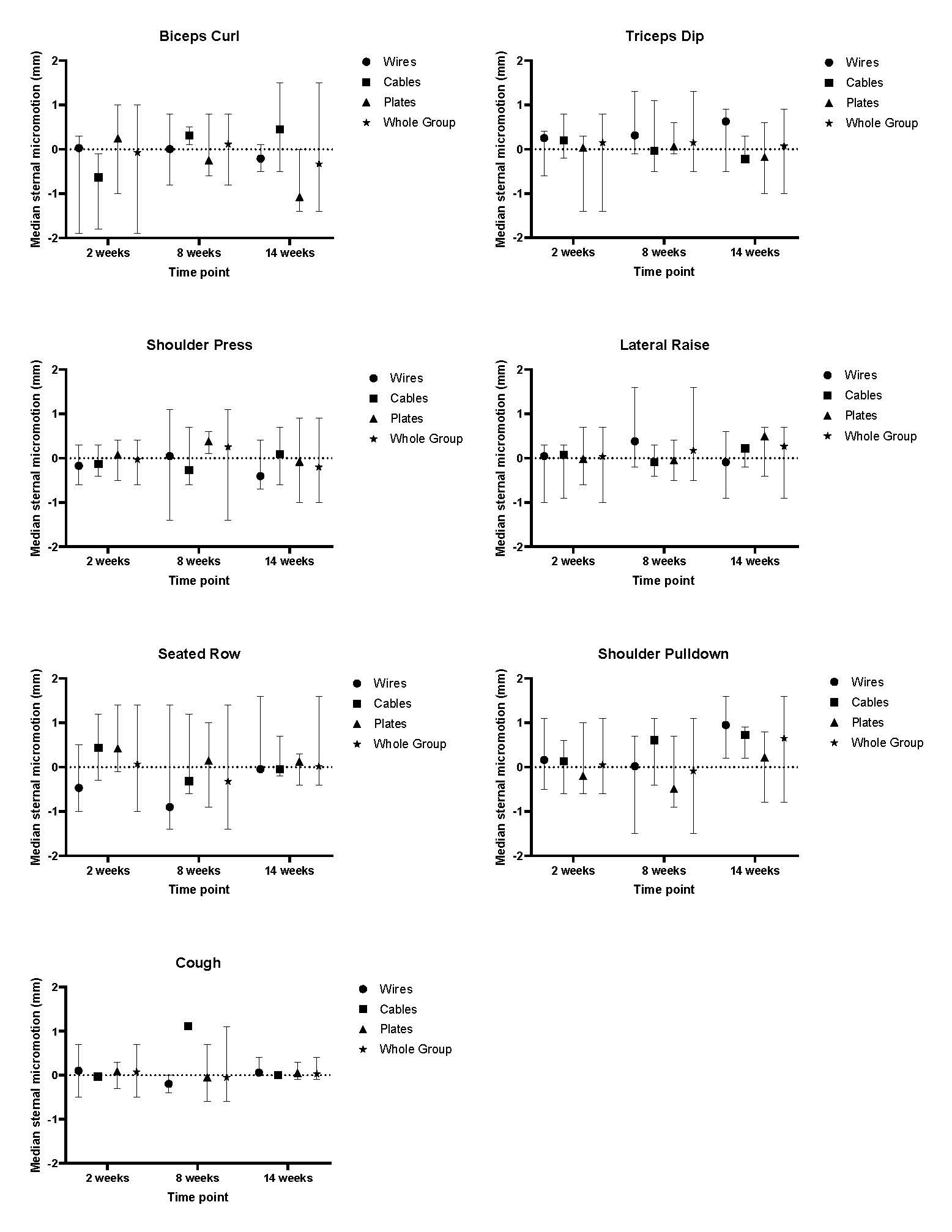

Supplement: Supplementary_Fig_3_pzac056 [file supplementary_fig_3_pzac056.jpeg]

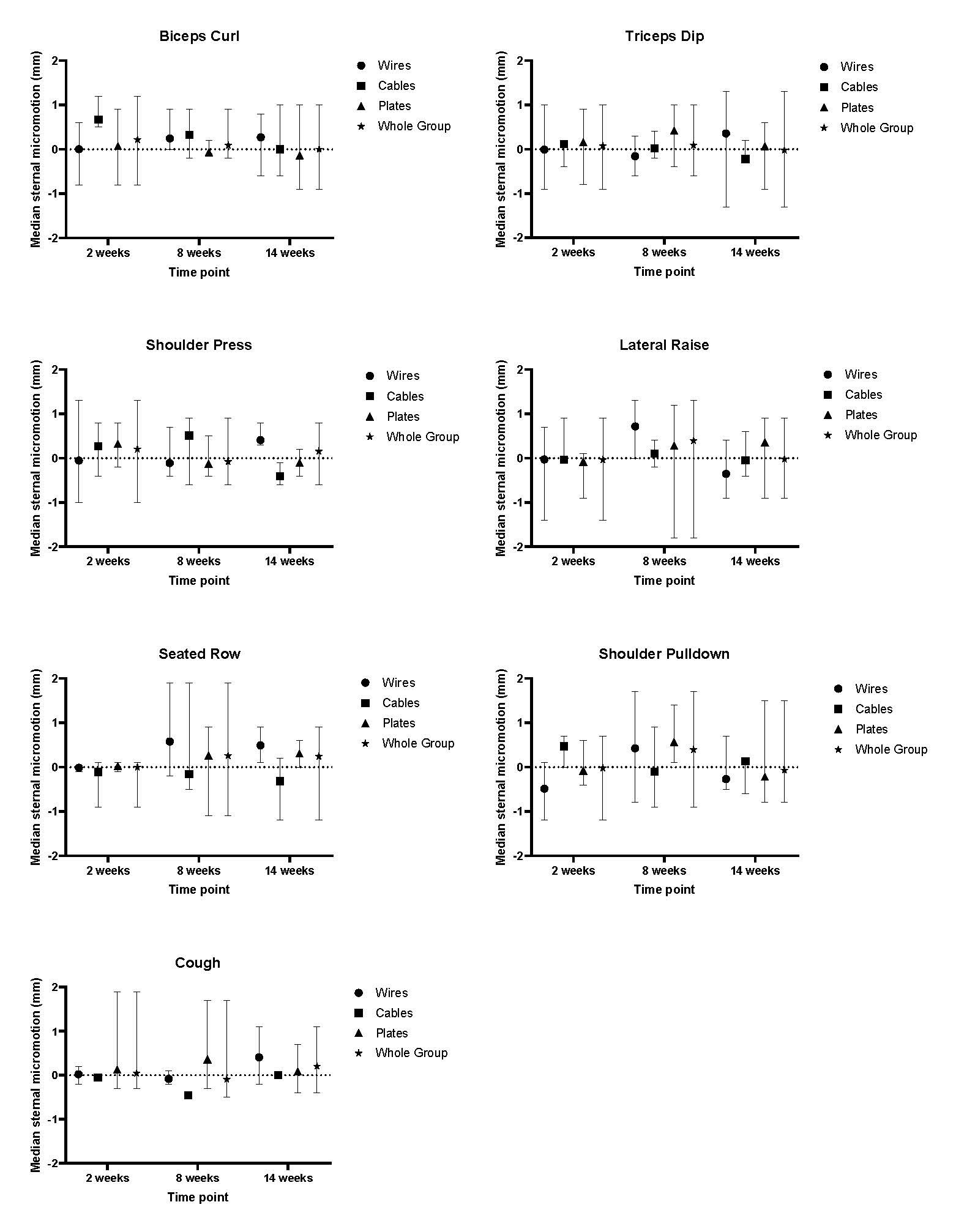

Supplement: Supplementary_Fig_4_pzac056 [file supplementary_fig_4_pzac056.jpeg]
