# Supplementary material for: SAfety and Feasibility of EArly Resistance Training After Median Sternotomy: The SAFE-ARMS Study
Source: Phys Ther. 2022 May 13;102(7):pzac056. doi: 10.1093/ptj/pzac056 (PMC9351378; doi:10.1093/ptj/pzac056)
Supplement: Supplementary_Table_1_pzac056 [file supplementary_table_1_pzac056.docx]

**Supplementary Table 1.** Median sternal micromotion at 2-, 8- and 14- weeks postoperatively, according to closure mechanism

| **Mid-sternum lateral micromotion** | | | | | | | | | | | | |
| --- | --- | --- | --- | --- | --- | --- | --- | --- | --- | --- | --- | --- |
|  | **Wires** | | | **Cables** | | | **Plates** | | | **Whole Group data** | | |
| **Exercise** | **2 weeks (n=6)** | **8 weeks (n=6)** | **14 weeks (n=5)** | **2 weeks (n=4)** | **8 weeks (n=4)** | **14 weeks (n=3)** | **2 weeks (n=6)** | **8 weeks (n=4)** | **14 weeks (n=4)** | **2 weeks (n=16)** | **8 weeks (n=14)** | **14 weeks (n=12)** |
| **Cough** | 0.02  (-0.1-0.8; n=4) | 1.01  (0.5-1.5; n=2) | -0.06  (-0.2-1.3; n=3) | 0.18 (0.18; n=1) | 3.23 (3.2; n=1) | 1.70 (1.7; n=1) | 0.50  (-1.6-2.5; n=6) | 0.79  (-1.5-2.5; n=4) | 0.28  (-0.2-2.6; n=4) | 0.15  (-1.6-2.5; n=11) | 0.81  (-1.5-3.2; n=7) | 0.28  (-0.2-2.6; n=8) |
| **Biceps curl** | 0.43  (0.10-1.2) | 1.05 (0.4-1.5) | -0.13  (-0.5-1.7) | 1.00  (-1.0-1.2) | 0.81  (-0.1-0.8) | 1.89 (1.3-1.9) | 0.72  (-1.4-1.4) | 0.57  (-0.2-1.8) | 0.90  (-0.6-1.9) | 0.72  (-1.4-1.4) | 0.83  (-0.2-1.8) | 1.30  (-0.6-1.9) |
| **Triceps dip** | 0.42  (-0.2-1.9) | 0.02 (-0.4-0.7) | 1.65  (-0.6-2.0) | 0.50  (-0.8-1.2) | 1.05 (0.4-1.5) | 0.91 (0.1-1.0) | 0.54  (-0.6-1.7) | 1.01  (-1.3-1.7) | 0.77 (0.2-1.4) | 0.42  (-0.8-1.9) | 0.65  (-1.3-1.7) | 0.85  (-0.6-2.0) |
| **Shoulder press** | 0.51  (0.1-1.4) | 0.81 (0.0-1.7) | 1.30  (-0.2-1.6) | 0.98  (-0.1-1.6) | 0.29  (-0.1-1.5) | 0.76 (0.3-1.0) | 0.81  (-1.3-1.6) | 0.46  (-0.9-1.5) | 0.21 (0.1-1.5) | 0.77  (-1.3-1.6) | 0.54  (-0.9-1.7) | 0.55  (-0.2-1.6) |
| **Lateral raise** | -0.14  (-1.3-0.9) | 0.58  (-0.2-1.6) | 1.12  (-0.4-1.4) | 0.69  (-0.6-1.4) | 0.22  (-0.1-0.9) | 1.61 (0.2-1.7) | 0.95  (-0.3-1.5) | 1.47  (-0.4-1.9) | 0.81 (0.3-1.7) | 0.64  (-1.3-1.5) | 0.58  (-0.4-1.9) | 1.05  (-0.4-1.7) |
| **Seated row** | 0.55  (-1.8-1.8) | 1.13  (-0.8-1.5) | 0.27  (-0.1-1.4) | 1.02 (0.1-1.3) | 1.44  (-0.5-1.7) | 1.22 (0.0-1.4) | 0.07  (-0.8-1.3) | 0.54 (0.0-1.7) | 1.46 (0.2-1.6) | 0.54  (-1.8-1.8) | 1.13  (-0.8-1.7) | 1.08  (-0.1-1.6) |
| **Shoulder pulldown** | 0.29  (-0.2-1.0) | 0.78  (-0.3-1.4) | -0.54  (-1.1-2.0) | 0.68 (0.0-1.3) | 0.11  (-1.3-1.5) | 0.05  (-0.6-0.9) | 0.32  (-0.2-0.8) | 0.62 (0.4-1.0) | 0.86 (0.3-1.2) | 0.33  (-0.2-1.3) | 0.49  (-1.3-1.5) | 0.37  (-1.1-2.0) |
| **Lower-sternum lateral micromotion** | | | | | | | | | | | | |
|  | **Wires** | | | **Cables** | | | **Plates** | | | **Whole Group data** | | |
| **Exercise** | **2 weeks (n=6)** | **8 weeks (n=6)** | **14 weeks (n=5)** | **2 weeks (n=4)** | **8 weeks (n=4)** | **14 weeks (n=3)** | **2 weeks (n=6)** | **8 weeks (n=4)** | **14 weeks (n=4)** | **2 weeks (n=16)** | **8 weeks (n=14)** | **14 weeks (n=12)** |
| **Cough** | -0.14  (-1.0-1.0; n=4) | 0.73 (0.7-0.7; n=2) | -0.14  (-1.0-0.6; n=3) | 1.77 (1.8; n=1) | 2.39 (2.4; n=1) | 1.62 (1.6; n=1) | 0.66 (0.1-2.9; n=6) | 0.65  (-0.9-2.1; n=4) | 1.10  (-0.1-1.5; n=4) | 0.63  (-1.0-2.9; n=11) | 0.74  (-0.9-2.4; n=7) | 0.74  (-1.0-1.6; n=8) |
| **Biceps curl** | 0.99  (-0.6-1.7) | 0.72  (-0.1-1.5) | 1.35  (-0.8-1.5) | 0.60 (0.1-1.2) | 0.23  (-0.6-1.8) | 0.90  (-0.4-1.3) | 0.81  (-1.1-1.7) | 0.50 (0.0-1.1) | 1.59 (1.1-2.0) | 0.80  (-1.1-1.7) | 0.68  (-0.6-1.8) | 1.33  (-0.8-2.0) |
| **Triceps dip** | 0.60  (-0.9-1.6) | 0.61  (-1.6-1.8) | 1.47  (-0.4-1.7) | 0.83  (-0.2-1.6) | 0.99 (0.6-1.4) | 1.17  (-0.1-1.7) | 0.51 (0.3-2.0) | 0.91 (0.3-2.0) | 1.07 (0.6-1.7) | 0.57  (-0.9-2.0) | 0.83  (-1.6-2.0) | 1.26  (-0.4-1.7) |
| **Shoulder press** | -0.21  (-1.5-1.4) | 1.04 (0.7-1.8) | 0.91 (0.0-1.7) | 1.19 (0.5-1.5) | 0.48  (-0.2-1.7) | 0.77  (-0.2-1.8) | 0.74  (-1.5-1.7) | 0.03  (-1.8-0.2) | 1.11 (0.7-1.7) | 0.63  (-1.5-1.7) | 0.65  (-1.8-1.8) | 0.94  (-0.2-1.8) |
| **Lateral raise** | 0.18  (-0.5-0.6) | 0.94  (-0.4-1.2) | 1.21  (-0.5-1.6) | 0.10  (-0.2-1.0) | -0.35  (-0.6-0.6) | 1.06  (-1.0 -1.2) | 0.71  (-0.3-1.5) | 0.10  (-1.3-0.6) | 1.29 (0.4-1.4) | 0.32  (-0.5-1.5) | 0.10  (-1.3-1.2) | 1.21  (-1.0-1.6) |
| **Seated row** | 0.49  (-1.1-1.4) | 0.23  (-0.4-1.2) | 1.26  (-0.3-1.8) | 0.82  (-1.7-1.5) | 0.02  (-0.1-1.1) | 0.00  (-0.2-0.7) | 0.69  (-0.5-1.6) | 0.12  (-0.3-0.3) | 0.63 (0.4-1.3) | 0.67  (-1.7-1.6) | 0.14  (-0.4-1.2) | 0.77  (-0.3-1.8) |
| **Shoulder pulldown** | 0.33 (0.0-1.1) | -0.29  (-0.4-0.3) | 0.40  (-0.3-1.9) | 0.79 (0.0-1.4) | -0.16  (-1.5-0.9) | 0.22  (-0.1-0.9) | 0.85 (0.0-1.2) | 0.07  (-0.4-1.8) | 0.64 (0.3-1.2) | 0.75 (0.0-1.4) | -0.22  (-1.5-1.8) | 0.46  (-0.3-1.9) |
| **Mid-sternum anterior-posterior micromotion** | | | | | | | | | | | | |
|  | **Wires** | | | **Cables** | | | **Plates** | | | **Whole Group data** | | |
| **Exercise** | **2 weeks (n=6)** | **8 weeks (n=6)** | **14 weeks (n=5)** | **2 weeks (n=4)** | **8 weeks (n=4)** | **14 weeks (n=3)** | **2 weeks (n=6)** | **8 weeks (n=4)** | **14 weeks (n=4)** | **2 weeks (n=16)** | **8 weeks (n=14)** | **14 weeks (n=12)** |
| **Cough** | 0.11  (-0.5-0.7; n=4) | -0.20  (-0.4-0.0; n=2) | 0.06 (0.0-0.4; n=3) | -0.03 (0.0; n=1) | 1.11 (1.1; n=1) | 0.00 (0.0; n=1) | 0.08  (-0.3-0.3; n=6) | -0.05  (-0.6-0.7; n=4) | 0.05  (-0.1-0.3; n=4) | 0.08  (-0.5-0.7; n=11) | -0.05  (-0.6-1.1; n=7) | 0.03  (-0.1-0.4; n=8) |
| **Biceps curl** | 0.03  (-1.9-0.3) | 0.00  (-0.8-0.8) | -0.21  (-0.5-0.1) | -0.63  (-1.8**–** -0.1) | 0.32 (0.1-0.5) | 0.45  (-0.5-1.5) | 0.25  (-1.0-1.0) | -0.25  (-0.6-0.8) | -1.08  (-1.4-0.0) | -0.07  (-1.9-1.0) | 0.12  (-0.8-0.8) | -0.33  (-1.4-1.5) |
| **Triceps dip** | 0.25  (-0.6-0.4) | 0.31  (-0.1-1.3) | 0.63  (-0.5-0.9) | 0.20  (-0.2-0.8) | -0.04  (-0.5-1.1) | -0.22  (-0.3-0.3) | 0.04  (-1.4-0.3) | 0.07  (-0.1-0.6) | -0.18  (-1.0-0.6) | 0.15  (-1.4-0.8) | 0.15  (-0.5-1.3) | 0.07  (-1.0-0.9) |
| **Shoulder press** | -0.17  (-0.6-0.3) | 0.05  (-1.4-1.1) | -0.40  (-0.7-0.4) | -0.14  (-0.4-0.3) | -0.27  (-0.6-0.7) | 0.09  (-0.6-0.7) | 0.07  (-0.5-0.4) | 0.38 (0.1-0.6) | -0.08 (-1.0-0.9) | -0.04  (-0.6-0.4) | 0.25  (-1.4-1.1) | -0.20  (-1.0-0.9) |
| **Lateral raise** | 0.04  (-1.0-0.3) | 0.38  (-0.2-1.6) | -0.09  (-0.9-0.6) | 0.08  (-0.9-0.3) | -0.09  (-0.4-0.3) | 0.22  (-0.2-0.3) | -0.02  (-0.6-0.7) | -0.05  (-0.5-0.4) | 0.50  (-0.4-0.7) | 0.04  (-1.0-0.7) | 0.18  (-0.5-1.6) | 0.27  (-0.9-0.7) |
| **Seated row** | -0.47  (-1.0-0.5) | -0.90  (-1.4-1.4) | -0.05  (-0.1-1.6) | 0.43  (-0.3-1.2) | -0.32  (-0.6-1.2) | -0.05  (-0.2-0.7) | 0.42  (-0.1-1.4) | 0.15  (-0.9-1.0) | 0.12  (-0.4-0.3) | 0.07  (-1.0-1.4) | -0.32  (-1.4-1.4) | 0.02  (-0.4-1.6) |
| **Shoulder pulldown** | 0.17  (-0.5-1.1) | 0.02  (-1.5-0.7) | 0.95 (0.2-1.6) | 0.13  (-0.6-0.6) | 0.61  (-0.4-1.1) | 0.72 (0.2-0.9) | -0.20  (-0.6-1.0) | -0.49  (-0.9-0.7) | 0.22  (-0.8-0.8) | 0.05  (-0.6-1.1) | -0.09  (-1.5-1.1) | 0.65  (-0.8-1.6) |
| **Lower-sternum anterior-posterior micromotion** | | | | | | | | | | | | |
|  | **Wires** | | | **Cables** | | | **Plates** | | | **Whole Group data** | | |
| **Exercise** | **2 weeks (n=6)** | **8 weeks (n=6)** | **14 weeks (n=5)** | **2 weeks (n=4)** | **8 weeks (n=4)** | **14 weeks (n=3)** | **2 weeks (n=6)** | **8 weeks (n=4)** | **14 weeks (n=4)** | **2 weeks (n=16)** | **8 weeks (n=14)** | **14 weeks (n=12)** |
| **Cough** | 0.02  (-0.2-0.2; n=4) | -0.08  (-0.2-0.1; n=2) | 0.41  (-0.2-1.1; n=3) | -0.06  (-0.1; n=1) | -0.46  (-0.5; n=1) | 0.00 (0.0; n=1) | 0.13  (-0.3-1.9; n=6) | 0.36  (-0.3-1.7; n=4) | 0.08  (-0.4-0.7; n=4) | 0.04  (-0.3-1.9; n=11) | -0.09  (-0.5-1.7; n=7) | 0.20  (-0.4-1.1; n=8) |
| **Biceps curl** | 0.00  (-0.8-0.6) | 0.25 (0.0-0.9) | 0.27  (-0.6-0.8) | 0.67 (0.5-1.2) | 0.33  (-0.2-0.9) | 0.00  (-0.6-1.0) | 0.07  (-0.8-0.9) | -0.07  (-0.1-0.2) | -0.14  (-0.9-1.0) | 0.22  (-0.8-1.2) | 0.09  (-0.2-0.9) | 0.00  (-0.9-1.0) |
| **Triceps dip** | -0.01  (-0.9-1.0) | -0.16  (-0.6-0.3) | 0.36  (-1.3-1.3) | 0.11  (-0.4-0.1) | 0.02  (-0.2-0.4) | -0.22  (-0.3-0.2) | 0.16  (-0.8-0.9) | 0.42  (-0.4-1.0) | 0.06  (-0.9-0.6) | 0.08  (-0.9-1.0) | 0.09  (-0.6-1.0) | -0.02  (-1.3-1.3) |
| **Shoulder press** | -0.05  (-1.0-1.3) | -0.11  (-0.4-0.7) | 0.41 (0.3-0.8) | 0.27  (-0.4-0.8) | 0.51  (-0.6-0.9) | -0.40  (-0.6**–** 0.1) | 0.32  (-0.2-0.8) | -0.13  (-0.4-0.5) | -0.10  (-0.4-0.2) | 0.20  (-1.0-1.3) | -0.08  (-0.6-0.9) | 0.15  (-0.6-0.8) |
| **Lateral raise** | -0.03  (-1.4-0.7) | 0.72 (0.0-1.3) | -0.36  (-0.9-0.4) | -0.04  (-0.1-0.9) | 0.11  (-0.2-0.4) | -0.05  (-0.4-0.6) | -0.08  (-0.9-0.1) | 0.28  (-1.8-1.2) | 0.36  (-0.9-0.9) | -0.04  (-1.4-0.9) | 0.39  (-1.8-1.3) | -0.02  (-0.9-0.9) |
| **Seated row** | -0.01  (-0.1-0.0) | 0.58  (-0.2-1.9) | 0.49 (0.1-0.9) | -0.11 (-0.9-0.1) | -0.16  (-0.5-1.9) | -0.32  (-1.2-0.2) | 0.02  (-0.1-0.1) | 0.26  (-1.1-0.9) | 0.31 (0.0-0.6) | 0.00 (0.9-0.1) | 0.26  (-1.1-1.9) | 0.24  (-1.2-0.9) |
| **Shoulder pulldown** | -0.49  (-1.2-0.1) | 0.43  (-0.8-1.7) | -0.27  (-0.5-0.7) | 0.47 (0.0-0.7) | -0.10  (-0.9-0.9) | 0.14  (-0.6-0.2) | -0.08  (-0.4-0.6) | 0.57 (0.1-1.4) | -0.22  (-0.8-1.5) | -0.02  (-1.2-0.7) | 0.40  (-0.9-1.7) | -0.07  (-0.8-1.5) |
