# Supplementary material for: SAfety and Feasibility of EArly Resistance Training After Median Sternotomy: The SAFE-ARMS Study
Source: Phys Ther. 2022 May 13;102(7):pzac056. doi: 10.1093/ptj/pzac056 (PMC9351378; doi:10.1093/ptj/pzac056)
Supplement: Supplement_A_pzac056 [file supplement_a_pzac056.docx]

**Supplement A.** Standardised body position and movements during each activity for ultrasound image obtainment

| **Activity** | **Seated position** | **Upper limb start position**  ***(start of concentric phase)*** | **Upper limb end position**  ***(start of eccentric phase)*** |
| --- | --- | --- | --- |
| Cough | 90-100⁰ hip flexion  90-100⁰ knee flexion  Feet flat on floor | N/A | N/A |
| Biceps curl | 90-100⁰ flexion  10⁰ trunk flexion | Underhand grip 55⁰ shoulder extension  0-10⁰ elbow flexion | 55⁰ shoulder flexion  125-135⁰ elbow flexion |
| Triceps dip | 90-100⁰ hip flexion  15⁰ trunk flexion | 45⁰ shoulder extension  45⁰ elbow flexion | neutral shoulder position (0⁰)  0-10⁰ elbow flexion |
| Shoulder press | 90-110⁰ hip flexion  Back in a neutral position (0⁰) | 40⁰ shoulder abduction  125⁰ elbow flexion | 0-10⁰ elbow flexion  130-160⁰ shoulder abduction |
| Shoulder pulldown | 90-110⁰ hip flexion  15⁰ trunk flexion | 150⁰ shoulder flexion  0-5⁰ elbow flexion | neutral shoulder position (0⁰)  95⁰ elbow flexion |
| Seated row | 90-110⁰ hip flexion  10⁰ trunk flexion  Feet flat on the floor | 90⁰ shoulder flexion  0⁰ elbow extension | 0-10⁰ shoulder extension  80-95⁰ elbow flexion |
| Lateral raise | 90⁰ hip flexion  Back neutral position (0⁰) | neutral shoulder position (0⁰)  85-90⁰ elbow flexion | 85-95⁰ shoulder abduction  85-90⁰ elbow flexion |
