# Supplementary material for: SAfety and Feasibility of EArly Resistance Training After Median Sternotomy: The SAFE-ARMS Study
Source: Phys Ther. 2022 May 13;102(7):pzac056. doi: 10.1093/ptj/pzac056 (PMC9351378; doi:10.1093/ptj/pzac056)
Supplement: Supplement_B_pzac056 [file supplement_b_pzac056.zip › Supplement_B_pzac056.docx]

**Supplement B.** Upper limb exercises and instructions to participants

| **Activity** | **Exercise instructions to participants** | **Upper limb start position**  ***(start of concentric phase)*** | **Upper limb end position**  ***(start of eccentric phase)*** |
| --- | --- | --- | --- |
| Biceps curl | In a seated position, grip the handholds in front with straight arms and an underhand grip. Lean forward slightly with chest firmly against the chest pad. Slowly bring your hands up toward your shoulders, bending at the elbows for a count of 4 seconds. Hold this position for a count of 2 seconds, before returning to the start position for a count of 4 seconds. | **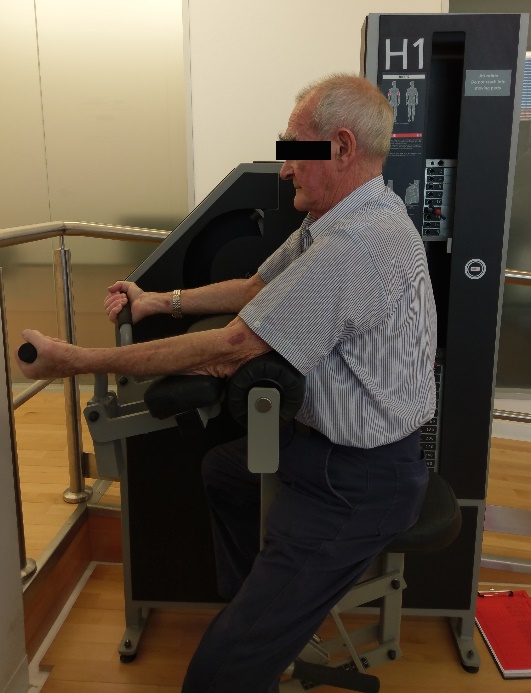** | 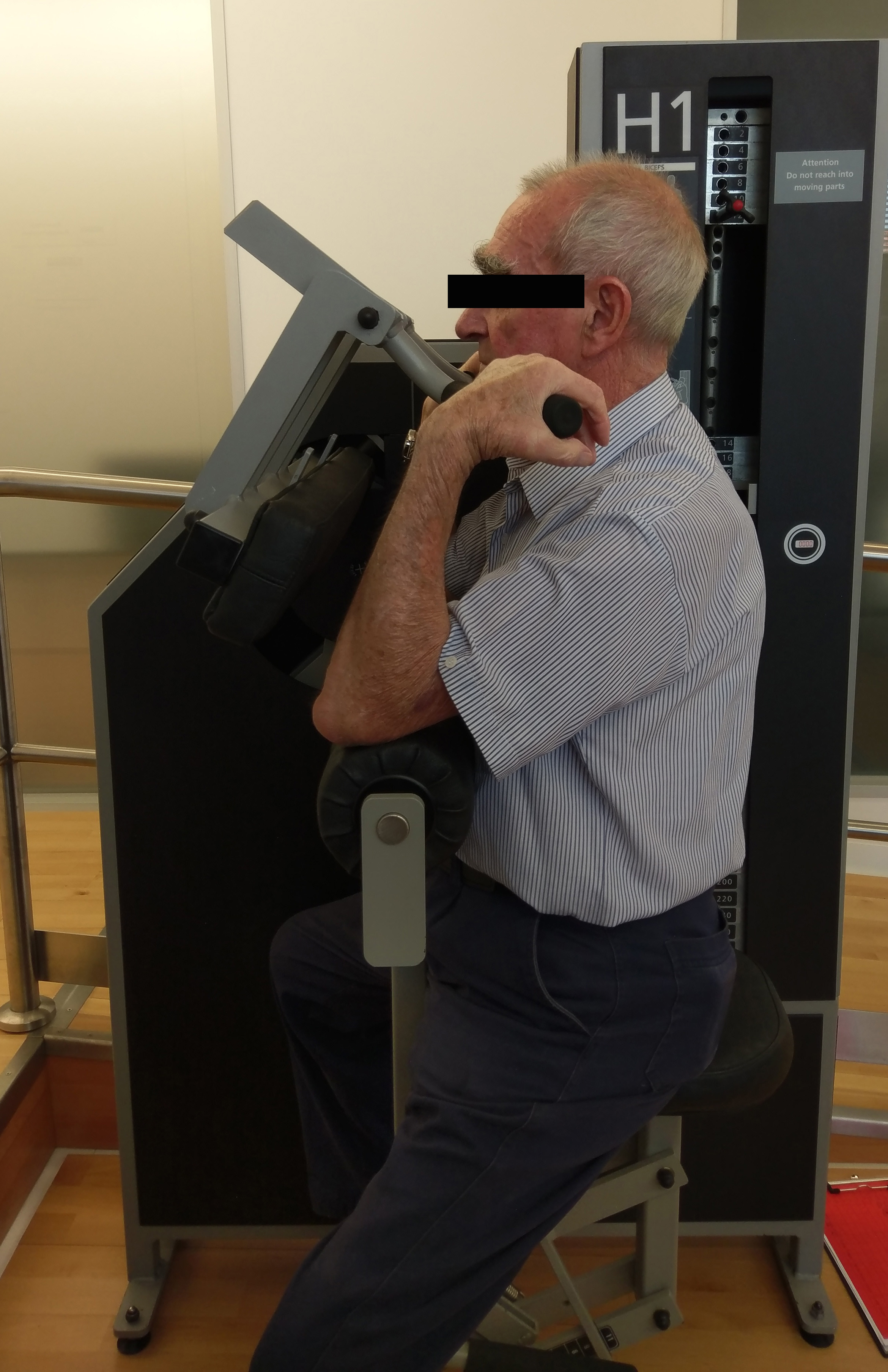 |
| Triceps dip | In a seated position, grip the handholds 5cm from the top with elbows bent. Lean forward slightly. Slowly push the handles down for a count of 4 seconds, straightening the elbows. Hold this position for a count of 2 seconds, before returning to the start position for a count of 4 seconds. | **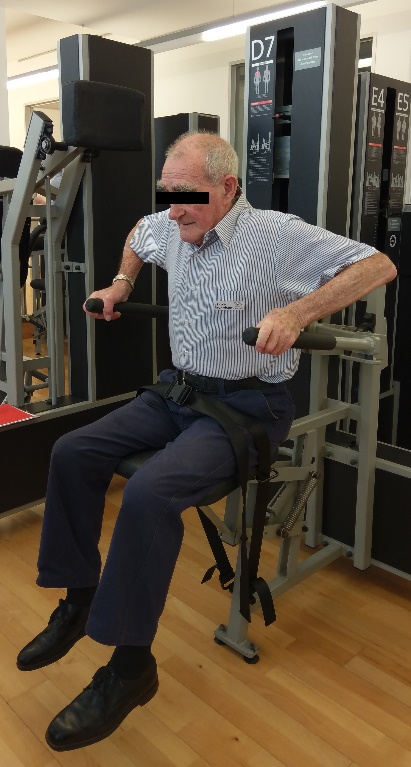** | **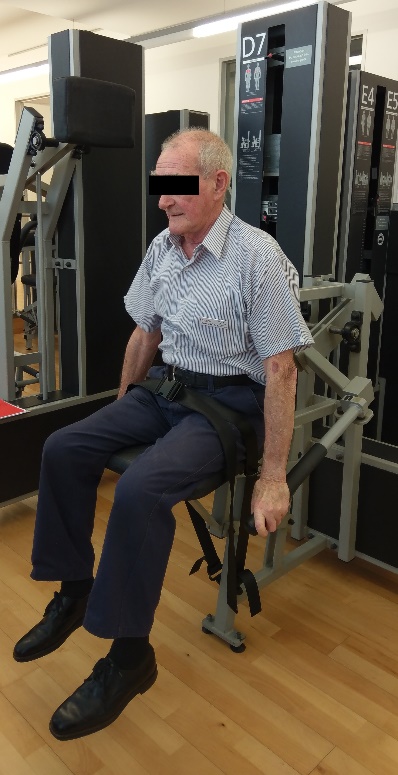** |
| Shoulder press | In a seated position with your back pressed against the back rest in a neutral position, grip the front handholds with elbows bent. Slowly straighten the elbows, lifting the arms overhead for a count of 4 seconds. Hold this position for a count of 2 seconds, before returning to the start position for a count of 4 seconds. | 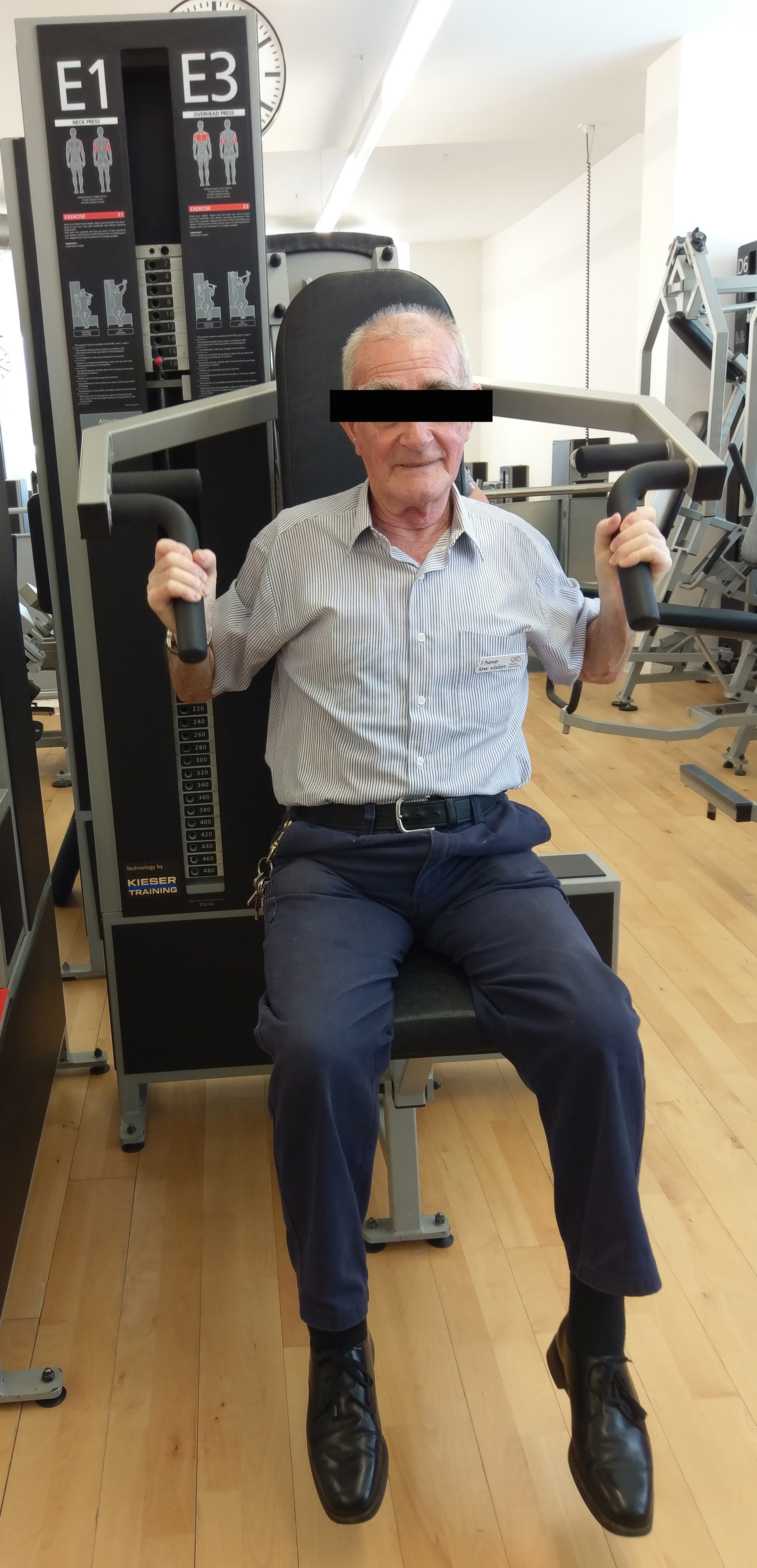 | **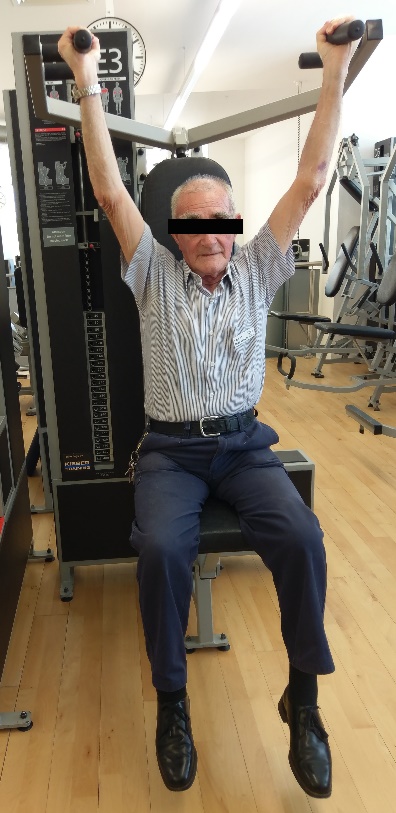** |
| Shoulder pulldown | In a seated position with fingertips just touching the handholds overhead. Lean forward slightly. Slowly pull the handles down for a count of 4 seconds, bending at the elbows. Hold this position for a count of 2 seconds, before returning to the start position for a count of 4 seconds. | 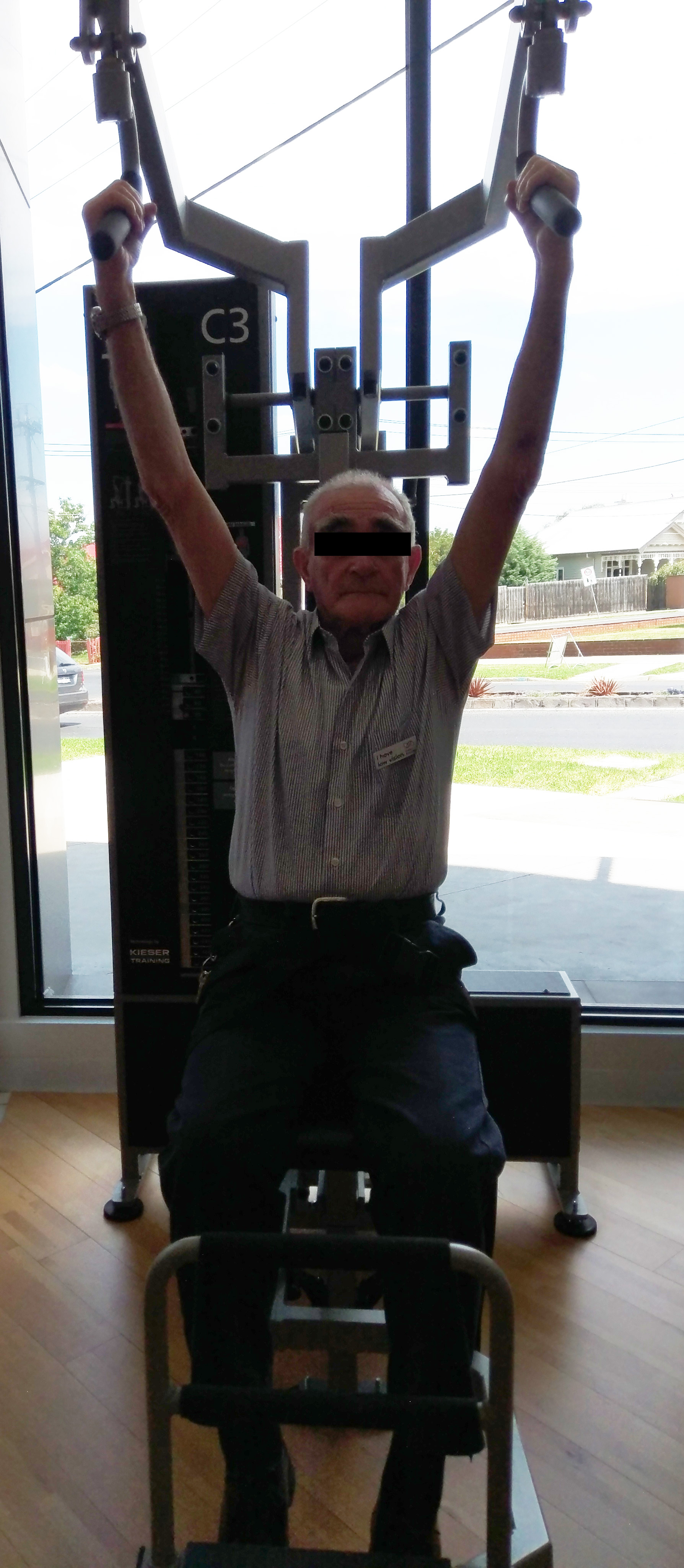 | **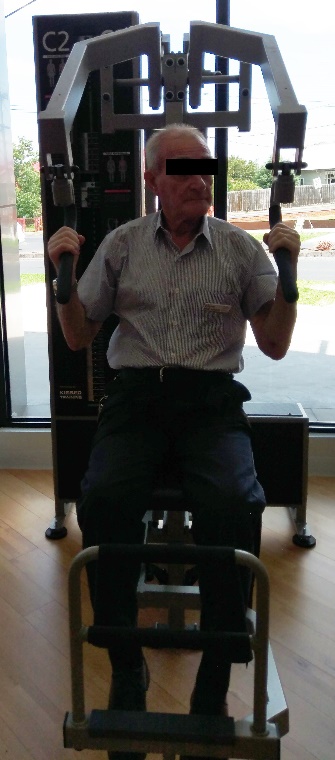** |
| Seated row | In a seated position with your feet flat on the floor and arms outstretched so fingertips just touch the handholds. Lean forward slightly with chest pressed against the chest pad. Slowly pull the handles toward you, bending at the elbows and keeping them close to the body for a count of 4 seconds. Hold this position for a count of 2 seconds, before returning to the start position for a count of 4 seconds. | **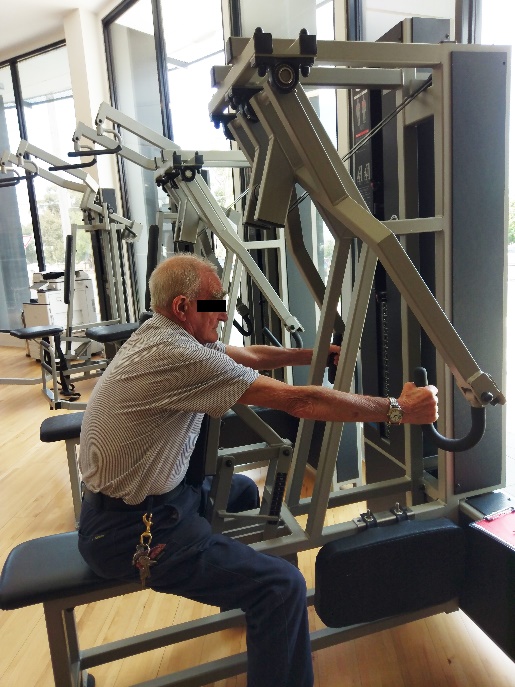** | **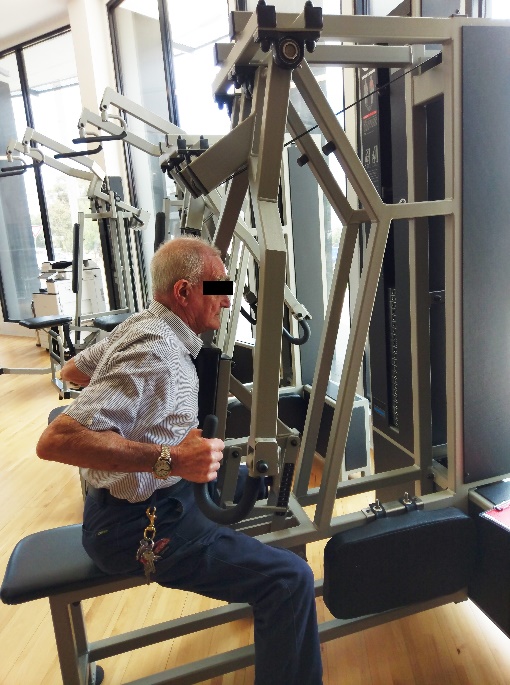** |
| Lateral raise | In a seated position with your back pressed against the back rest in a neutral position and elbows bent at the sides against the elbow pads. Slowly lift the shoulders out to the sides for a count of 4 seconds keeping the elbows pressed against the elbow pads. Hold this position for a count of 2 seconds, before returning to the start position for a count of 4 seconds. | 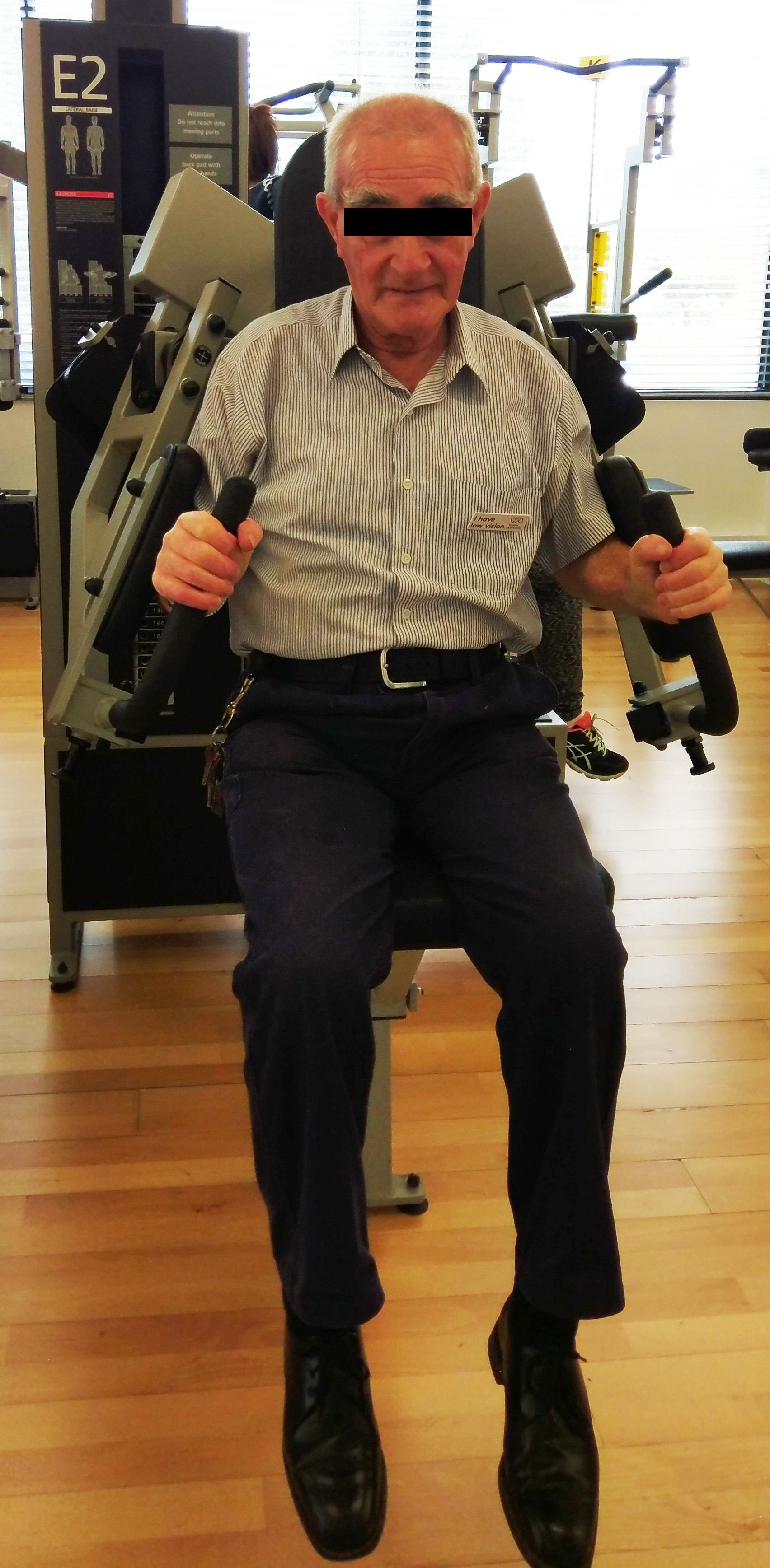 | **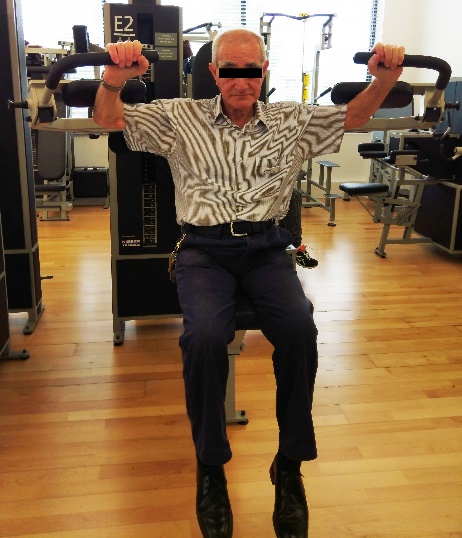** |
