# Supplementary material for: SAfety and Feasibility of EArly Resistance Training After Median Sternotomy: The SAFE-ARMS Study
Source: Phys Ther. 2022 May 13;102(7):pzac056. doi: 10.1093/ptj/pzac056 (PMC9351378; doi:10.1093/ptj/pzac056)
Supplement: Supplement_C_pzac056 [file supplement_c_pzac056.docx]

**Supplement C.** Sternal micromotion results, analysed according to sternal closure mechanism.

Mid-sternum lateral micromotion

At the mid-sternum (6cm from the sternal notch), patients who had cables as their mechanism of sternal closure/fixation exceeded the resistance training group median data more frequently than either the wires or plates group *(Supp. Table 1, Supp. Fig.1)*. The greatest variance from the median for each exercise occurred in the wires group at 14 weeks for the biceps curl (-0.13 [-0.5-1.7]), triceps dip (1.65 [-0.6-2.0]), shoulder press (1.30 [-0.2-1.6]), seated row (0.27 [-0.1-1.4]) and shoulder pulldown (-0.54 [-1.1-2.0]); in the cables group for the cough at 8 weeks (3.23 [3.2]); and, in the plate group for the lateral raise at 8 weeks (1.47 [-0.4-1.9]; *Supp. Table 1, Supp. Fig.1*).

Lower-sternum lateral micromotion

At the lower-sternum (10cm from the sternal notch), the number of occasions the resistance group median data was exceeded was comparable between the three mechanisms of sternal closure/fixation *(Supp. Table 1, Supp. Fig.2)*. The greatest variance from the median for each exercise occurred in the cables group for the triceps dip (0.83 [-0.2-1.6]) at 2 weeks, cough (2.39 [2.4]) and biceps curl (0.23 [-0.6-1.8]) at 8 weeks, and the seated row at 14 weeks (0.00 [-0.2-0.7]); and, in the wires group for the shoulder press (-0.21 [-1.5-1.4]) and shoulder pulldown (0.33 [0.0-1.1]) at 2 weeks, and lateral raise at 8 weeks (0.94 [-0.4-1.2]; *Supp. Table 1, Supp. Fig.2*).

Mid-sternum anterior-posterior micromotion

At the mid-sternum (6cm from the sternal notch), the number of occasions the resistance group median data was exceeded was comparable between the three mechanisms of sternal closure/fixation *(Supp. Table 1, Supp. Fig.3)*. The greatest variance from the median for each exercise occurred in the cables group at 8 weeks for the cough (1.11 [1.1]), shoulder press (-0.27 [-0.6-0.7]) and shoulder pulldown (0.61 [-0.4-1.1]), and biceps curl at 14 weeks (0.45 [-0.5-1.5]); and, in the wires group for the seated row at 8 weeks (-0.90 [-1.4-1.4]), and the triceps dip (0.63 [-0.5-0.9]) and lateral raise (-0.09 [-0.9-0.6]) at 14 weeks *(Supp Table 1, Supp. Fig.3*).

Lower-sternum anterior-posterior micromotion

At the lower-sternum (10cm from the sternal notch), patients who had wires as their mechanism of sternal closure/fixation exceeded the resistance training group median data more frequently than either the cables or plates group *(Supp. Table 1, Supp. Fig.4)*. The greatest variance from the median for each exercise occurred in the cables group for the biceps curl (0.67 [0.5-1.2]) at 2 weeks, shoulder press (0.51 [-0.6-0.9]) and shoulder pulldown (-0.10 [-0.9-0.9]) at 8 weeks, and the seated row at 14 weeks (-0.32 [-1.2-0.2]); in the plates group for the cough at 8 weeks (0.36 [-0.3-1.7]) and the lateral raise at 14 weeks (0.36 [-0.9-0.9]); and, in the wires group for the triceps dip at 14 weeks (0.36 [-1.3-1.3]; *Supp. Table 1, Supp. Fig.4*).

**Supplementary Table 1.** Median sternal micromotion at 2-, 8- and 14- weeks postoperatively, according to closure mechanism

| **Mid-sternum lateral micromotion** | | | | | | | | | | | | |
| --- | --- | --- | --- | --- | --- | --- | --- | --- | --- | --- | --- | --- |
|  | **Wires** | | | **Cables** | | | **Plates** | | | **Whole Group data** | | |
| **Exercise** | **2 weeks (n=6)** | **8 weeks (n=6)** | **14 weeks (n=5)** | **2 weeks (n=4)** | **8 weeks (n=4)** | **14 weeks (n=3)** | **2 weeks (n=6)** | **8 weeks (n=4)** | **14 weeks (n=4)** | **2 weeks (n=16)** | **8 weeks (n=14)** | **14 weeks (n=12)** |
| **Cough** | 0.02 (-0.1-0.8; n=4) | 1.01 (0.5-1.5; n=2) | -0.06 (-0.2-1.3; n=3) | 0.18 (0.18; n=1) | 3.23 (3.2; n=1) | 1.70 (1.7; n=1) | 0.50 (-1.6-2.5’ n=6) | 0.79 (-1.5-2.5; n=4) | 0.28 (-0.2-2.6; n=4) | 0.15 (-1.6-2.5; n=11) | 0.81 (-1.5-3.2; n=7) | 0.28 (-0.2-2.6; n=8) |
| **Biceps curl** | 0.43 (0.10-1.2) | 1.05 (0.4-1.5) | -0.13 (-0.5-1.7) | 1.00 (-1.0-1.2) | 0.81 (-0.1-0.8) | 1.89 (1.3-1.9) | 0.72 (-1.4-1.4) | 0.57 (-0.2-1.8) | 0.90 (-0.6-1.9) | 0.72 (-1.4-1.4) | 0.83 (-0.2-1.8) | 1.30 (-0.6-1.9) |
| **Triceps dip** | 0.42 (-0.2-1.9) | 0.02 (-0.4-0.7) | 1.65 (-0.6-2.0) | 0.50 (-0.8-1.2) | 1.05 (0.4-1.5) | 0.91 (0.1-1.0) | 0.54 (-0.6-1.7) | 1.01 (-1.3-1.7) | 0.77 (0.2-1.4) | 0.42 (-0.8-1.9) | 0.65 (-1.3-1.7) | 0.85 (-0.6-2.0) |
| **Shoulder press** | 0.51 (0.1-1.4) | 0.81 (0.0-1.7) | 1.30 (-0.2-1.6) | 0.98 (-0.1-1.6) | 0.29 (-0.1-1.5) | 0.76 (0.3-1.0) | 0.81 (-1.3-1.6) | 0.46 (-0.9-1.5) | 0.21 (0.1-1.5) | 0.77 (-1.3-1.6) | 0.54 (-0.9-1.7) | 0.55 (-0.2-1.6) |
| **Lateral raise** | -0.14 (-1.3-0.9) | 0.58 (-0.2-1.6) | 1.12 (-0.4-1.4) | 0.69 (-0.6-1.4) | 0.22 (-0.1-0.9) | 1.61 (0.2-1.7) | 0.95 (-0.3-1.5) | 1.47 (-0.4-1.9) | 0.81 (0.3-1.7) | 0.64 (-1.3-1.5) | 0.58 (-0.4-1.9) | 1.05 (-0.4-1.7) |
| **Seated row** | 0.55 (-1.8-1.8) | 1.13 (-0.8-1.5) | 0.27 (-0.1-1.4) | 1.02 (0.1-1.3) | 1.44 (-0.5-1.7) | 1.22 (0.0-1.4) | 0.07 (-0.8-1.3) | 0.54 (0.0-1.7) | 1.46 (0.2-1.6) | 0.54 (-1.8-1.8) | 1.13 (-0.8-1.7) | 1.08 (-0.1-1.6) |
| **Shoulder pulldown** | 0.29 (-0.2-1.0) | 0.78 (-0.3-1.4) | -0.54 (-1.1-2.0) | 0.68 (0.0-1.3) | 0.11 (-1.3-1.5) | 0.05 (-0.6-0.9) | 0.32 (-0.2-0.8) | 0.62 (0.4-1.0) | 0.86 (0.3-1.2) | 0.33 (-0.2-1.3) | 0.49 (-1.3-1.5) | 0.37 (-1.1-2.0) |
| **Lower-sternum lateral micromotion** | | | | | | | | | | | | |
|  | **Wires** | | | **Cables** | | | **Plates** | | | **Whole Group data** | | |
| **Exercise** | **2 weeks (n=6)** | **8 weeks (n=6)** | **14 weeks (n=5)** | **2 weeks (n=4)** | **8 weeks (n=4)** | **14 weeks (n=3)** | **2 weeks (n=6)** | **8 weeks (n=4)** | **14 weeks (n=4)** | **2 weeks (n=16)** | **8 weeks (n=14)** | **14 weeks (n=12)** |
| **Cough** | -0.14 (-1.0-1.0; n=4) | 0.73 (0.7-0.7; n=2) | -0.14 (-1.0-0.6; n=3) | 1.77 (1.8; n=1) | 2.39 (2.4; n=1) | 1.62 (1.6; n=1) | 0.66 (0.1-2.9; n=6) | 0.65 (-0.9-2.1; n=4) | 1.10 (-0.1-1.5; n=4) | 0.63 (-1.0-2.9; n=11) | 0.74 (-0.9-2.4; n=7) | 0.74 (-1.0-1.6; n=8) |
| **Biceps curl** | 0.99 (-0.6-1.7) | 0.72 (-0.1-1.5) | 1.35 (-0.8-1.5) | 0.60 (0.1-1.2) | 0.23 (-0.6-1.8) | 0.90 (-0.4-1.3) | 0.81 (-1.1-1.7) | 0.50 (0.0-1.1) | 1.59 (1.1-2.0) | 0.80 (-1.1-1.7) | 0.68 (-0.6-1.8) | 1.33 (-0.8-2.0) |
| **Triceps dip** | 0.60 (-0.9-1.6) | 0.61 (-1.6-1.8) | 1.47 (-0.4-1.7) | 0.83 (-0.2-1.6) | 0.99 (0.6-1.4) | 1.17 (-0.1-1.7) | 0.51 (0.3-2.0) | 0.91 (0.3-2.0) | 1.07 (0.6-1.7) | 0.57 (-0.9-2.0) | 0.83 (-1.6-2.0) | 1.26 (-0.4-1.7) |
| **Shoulder press** | -0.21 (-1.5-1.4) | 1.04 (0.7-1.8) | 0.91 (0.0-1.7) | 1.19 (0.5-1.5) | 0.48 (-0.2-1.7) | 0.77 (-0.2-1.8) | 0.74 (-1.5-1.7) | 0.03 (-1.8-0.2) | 1.11 (0.7-1.7) | 0.63 (-1.5-1.7) | 0.65 (-1.8-1.8) | 0.94 (-0.2-1.8) |
| **Lateral raise** | 0.18 (-0.5-0.6) | 0.94 (-0.4-1.2) | 1.21 (-0.5-1.6) | 0.10 (-0.2-1.0) | -0.35 (-0.6-0.6) | 1.06 (-1.0 -1.2) | 0.71 (-0.3-1.5) | 0.10 (-1.3-0.6) | 1.29 (0.4-1.4) | 0.32 (-0.5-1.5) | 0.10 (-1.3-1.2) | 1.21 (-1.0-1.6) |
| **Seated row** | 0.49 (-1.1-1.4) | 0.23 (-0.4-1.2) | 1.26 (-0.3-1.8) | 0.82 (-1.7-1.5) | 0.02 (-0.1-1.1) | 0.00 (-0.2-0.7) | 0.69 (-0.5-1.6) | 0.12 (-0.3-0.3) | 0.63 (0.4-1.3) | 0.67 (-1.7-1.6) | 0.14 (-0.4-1.2) | 0.77 (-0.3-1.8) |
| **Shoulder pulldown** | 0.33 (0.0-1.1) | -0.29 (-0.4-0.3) | 0.40 (-0.3-1.9) | 0.79 (0.0-1.4) | -0.16 (-1.5-0.9) | 0.22 (-0.1-0.9) | 0.85 (0.0-1.2) | 0.07 (-0.4-1.8) | 0.64 (0.3-1.2) | 0.75 (0.0-1.4) | -0.22 (-1.5-1.8) | 0.46 (-0.3-1.9) |
| **Mid-sternum anterior-posterior micromotion** | | | | | | | | | | | | |
|  | **Wires** | | | **Cables** | | | **Plates** | | | **Whole Group data** | | |
| **Exercise** | **2 weeks (n=6)** | **8 weeks (n=6)** | **14 weeks (n=5)** | **2 weeks (n=4)** | **8 weeks (n=4)** | **14 weeks (n=3)** | **2 weeks (n=6)** | **8 weeks (n=4)** | **14 weeks (n=4)** | **2 weeks (n=16)** | **8 weeks (n=14)** | **14 weeks (n=12)** |
| **Cough** | 0.11 (-0.5-0.7; n=4) | -0.20 (-0.4-0.0; n=2) | 0.06 (0.0-0.4; n=3) | -0.03 (0.0; n=1) | 1.11 (1.1; n=1) | 0.00 (0.0; n=1) | 0.08 (-0.3-0.3; n=6) | -0.05 (-0.6-0.7; n=4) | 0.05 (-0.1-0.3; n=4) | 0.08 (-0.5-0.7; n=11) | -0.05 (-0.6-1.1; n=7) | 0.03 (-0.1-0.4; n=8) |
| **Biceps curl** | 0.03 (-1.9-0.3) | 0.00 (-0.8-0.8) | -0.21 (-0.5-0.1) | -0.63 (-1.8**–** -0.1) | 0.32 (0.1-0.5) | 0.45 (-0.5-1.5) | 0.25 (-1.0-1.0) | -0.25 (-0.6-0.8) | -1.08 (-1.4-0.0) | -0.07 (-1.9-1.0) | 0.12 (-0.8-0.8) | -0.33 (-1.4-1.5) |
| **Triceps dip** | 0.25 (-0.6-0.4) | 0.31 (-0.1-1.3) | 0.63 (-0.5-0.9) | 0.20 (-0.2-0.8) | -0.04 (-0.5-1.1) | -0.22 (-0.3-0.3) | 0.04 (-1.4-0.3) | 0.07 (-0.1-0.6) | -0.18 (-1.0-0.6) | 0.15 (-1.4-0.8) | 0.15 (-0.5-1.3) | 0.07 (-1.0-0.9) |
| **Shoulder press** | -0.17 (-0.6-0.3) | 0.05 (-1.4-1.1) | -0.40 (-0.7-0.4) | -0.14 (-0.4-0.3) | -0.27 (-0.6-0.7) | 0.09 (-0.6-0.7) | 0.07 (-0.5-0.4) | 0.38 (0.1-0.6) | -0.08 (-1.0-0.9) | -0.04 (-0.6-0.4) | 0.25 (-1.4-1.1) | -0.20 (-1.0-0.9) |
| **Lateral raise** | 0.04 (-1.0-0.3) | 0.38 (-0.2-1.6) | -0.09 (-0.9-0.6) | 0.08 (-0.9-0.3) | -0.09 (-0.4-0.3) | 0.22 (-0.2-0.3) | -0.02 (-0.6-0.7) | -0.05 (-0.5-0.4) | 0.50 (-0.4-0.7) | 0.04 (-1.0-0.7) | 0.18 (-0.5-1.6) | 0.27 (-0.9-0.7) |
| **Seated row** | -0.47 (-1.0-0.5) | -0.90 (-1.4-1.4) | -0.05 (-0.1-1.6) | 0.43 (-0.3-1.2) | -0.32 (-0.6-1.2) | -0.05 (-0.2-0.7) | 0.42 (-0.1-1.4) | 0.15 (-0.9-1.0) | 0.12 (-0.4-0.3) | 0.07 (-1.0-1.4) | -0.32 (-1.4-1.4) | 0.02 (-0.4-1.6) |
| **Shoulder pulldown** | 0.17 (-0.5-1.1) | 0.02 (-1.5-0.7) | 0.95 (0.2-1.6) | 0.13 (-0.6-0.6) | 0.61 (-0.4-1.1) | 0.72 (0.2-0.9) | -0.20 (-0.6-1.0) | -0.49 (-0.9-0.7) | 0.22 (-0.8-0.8) | 0.05 (-0.6-1.1) | -0.09 (-1.5-1.1) | 0.65 (-0.8-1.6) |
| **Lower-sternum anterior-posterior micromotion** | | | | | | | | | | | | |
|  | **Wires** | | | **Cables** | | | **Plates** | | | **Whole Group data** | | |
| **Exercise** | **2 weeks (n=6)** | **8 weeks (n=6)** | **14 weeks (n=5)** | **2 weeks (n=4)** | **8 weeks (n=4)** | **14 weeks (n=3)** | **2 weeks (n=6)** | **8 weeks (n=4)** | **14 weeks (n=4)** | **2 weeks (n=16)** | **8 weeks (n=14)** | **14 weeks (n=12)** |
| **Cough** | 0.02 (-0.2-0.2; n=4) | -0.08 (-0.2-0.1; n=2) | 0.41 (-0.2-1.1; n=3) | -0.06 (-0.1; n=1) | -0.46 (-0.5; n=1) | 0.00 (0.0; n=1) | 0.13 (-0.3-1.9; n=6) | 0.36 (-0.3-1.7; n=4) | 0.08 (-0.4-0.7; n=4) | 0.04 (-0.3-1.9; n=11) | -0.09 (-0.5-1.7; n=7) | 0.20 (-0.4-1.1; n=8) |
| **Biceps curl** | 0.00 (-0.8-0.6) | 0.25 (0.0-0.9) | 0.27 (-0.6-0.8) | 0.67 (0.5-1.2) | 0.33 (-0.2-0.9) | 0.00 (-0.6-1.0) | 0.07 (-0.8-0.9) | -0.07 (-0.1-0.2) | -0.14 (-0.9-1.0) | 0.22 (-0.8-1.2) | 0.09 (-0.2-0.9) | 0.00 (-0.9-1.0) |
| **Triceps dip** | -0.01 (-0.9-1.0) | -0.16 (-0.6-0.3) | 0.36 (-1.3-1.3) | 0.11 (-0.4-0.1) | 0.02 (-0.2-0.4) | -0.22 (-0.3-0.2) | 0.16 (-0.8-0.9) | 0.42 (-0.4-1.0) | 0.06 (-0.9-0.6) | 0.08 (-0.9-1.0) | 0.09 (-0.6-1.0) | -0.02 (-1.3-1.3) |
| **Shoulder press** | -0.05 (-1.0-1.3) | -0.11 (-0.4-0.7) | 0.41 (0.3-0.8) | 0.27 (-0.4-0.8) | 0.51 (-0.6-0.9) | -0.40 (-0.6**– 0.1**) | 0.32 (-0.2-0.8) | -0.13 (-0.4-0.5) | -0.10 (-0.4-0.2) | 0.20 (-1.0-1.3) | -0.08 (-0.6-0.9) | 0.15 (-0.6-0.8) |
| **Lateral raise** | -0.03 (-1.4-0.7) | 0.72 (0.0-1.3) | -0.36 (-0.9-0.4) | -0.04 (-0.1-0.9) | 0.11 (-0.2-0.4) | -0.05 (-0.4-0.6) | -0.08 (-0.9-0.1) | 0.28 (-1.8-1.2) | 0.36 (-0.9-0.9) | -0.04 (-1.4-0.9) | 0.39 (-1.8-1.3) | -0.02 (-0.9-0.9) |
| **Seated row** | -0.01 (-0.1-0.0) | 0.58 (-0.2-1.9) | 0.49 (0.1-0.9) | -0.11 (-0.9-0.1) | -0.16 (-0.5-1.9) | -0.32 (-1.2-0.2) | 0.02 (-0.1-0.1) | 0.26 (-1.1-0.9) | 0.31 (0.0-0.6) | 0.00 (0.9-0.1) | 0.26 (-1.1-1.9) | 0.24 (-1.2-0.9) |
| **Shoulder pulldown** | -0.49 (-1.2-0.1) | 0.43 (-0.8-1.7) | -0.27 (-0.5-0.7) | 0.47 (0.0-0.7) | -0.10 (-0.9-0.9) | 0.14 (-0.6-0.2) | -0.08 (-0.4-0.6) | 0.57 (0.1-1.4) | -0.22 (-0.8-1.5) | -0.02 (-1.2-0.7) | 0.40 (-0.9-1.7) | -0.07 (-0.8-1.5) |

**Supplementary Figure 1**. Changes in lateral motion at the sternal edges (mm) according to sternal closure mechanism (wires, cables or plates) during a biceps curl (A), triceps dip (B), shoulder press (C), lateral raise (D), seated row (E), shoulder pulldown (F) and cough (G) at the mid-sternum at 2-, 8- and 14- weeks post-operatively.


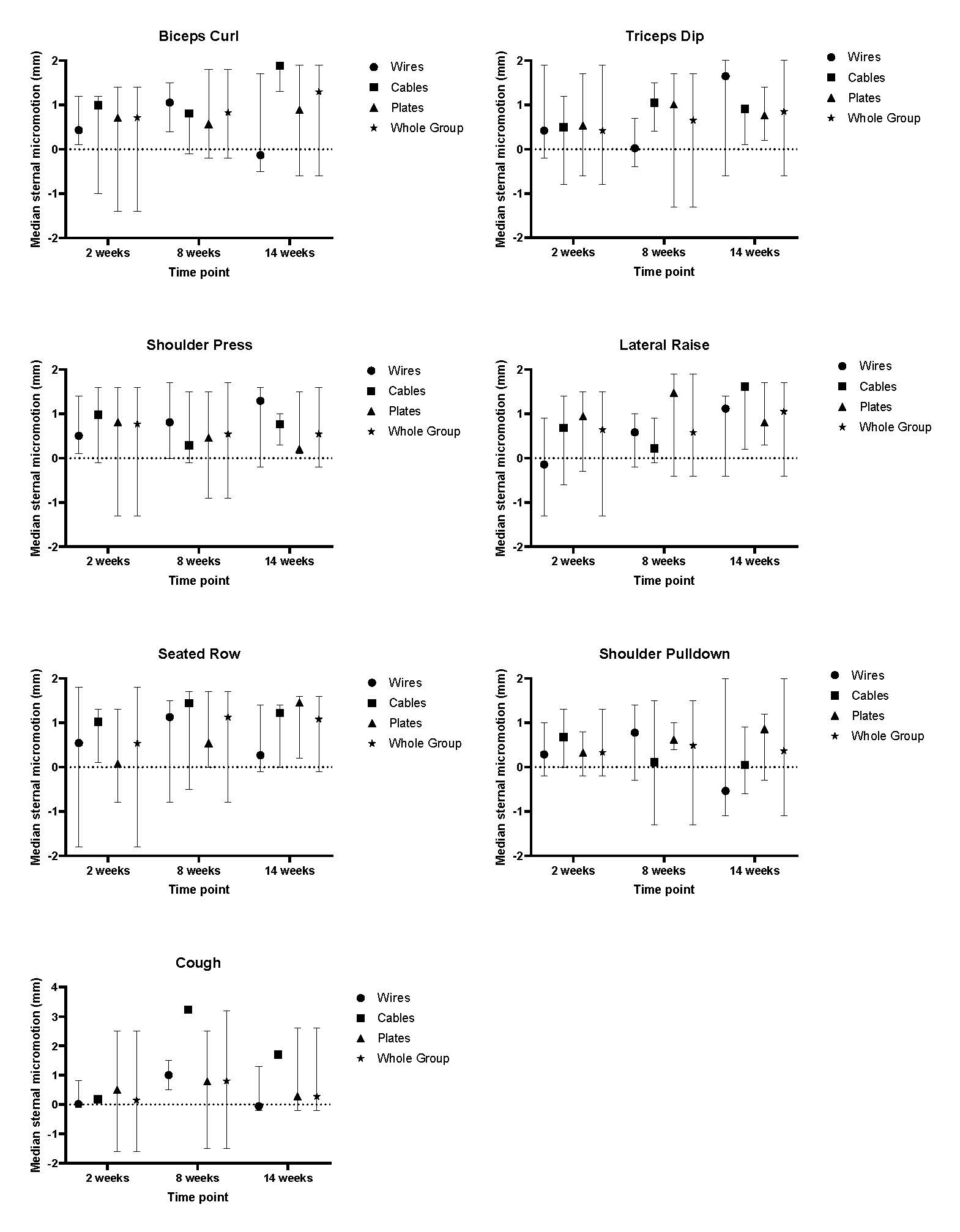


**A)**

**B)**

**C)**

**D)**

**E)**

**F)**

**G)**

**Supplementary Figure 2**. Changes in lateral motion at the sternal edges (mm) according to sternal closure mechanism (wires, cables or plates) during a biceps curl (A), triceps dip (B), shoulder press (C), lateral raise (D), seated row (E), shoulder pulldown (F) and cough (G) at the lower-sternum at 2-, 8- and 14- weeks post-operatively.


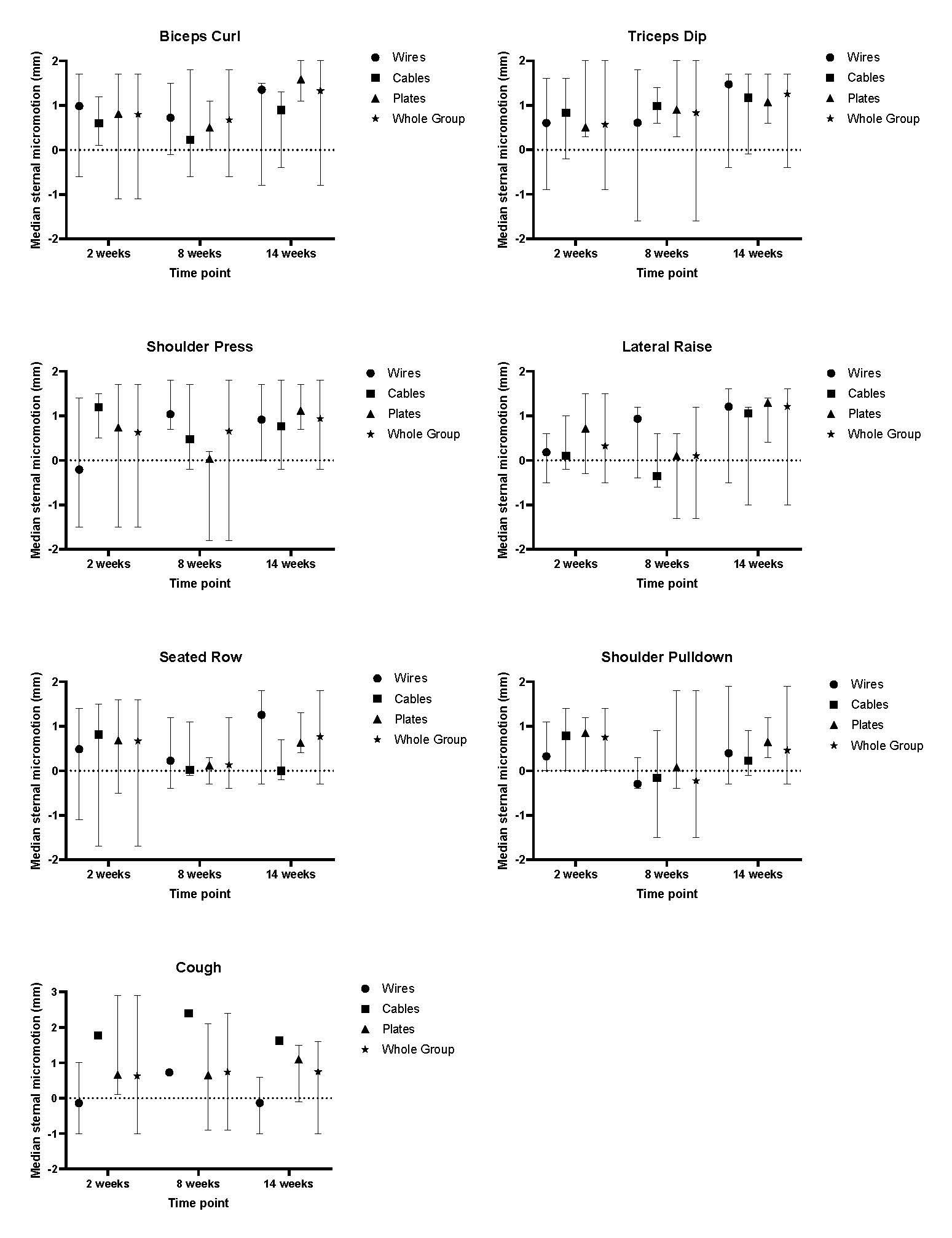


**A)**

**B)**

**C)**

**D)**

**E)**

**F)**

**G)**

**Supplementary Figure 3**. Changes in anterior-posterior motion at the sternal edges (mm) according to sternal closure mechanism (wires, cables or plates) during a biceps curl (A), triceps dip (B), shoulder press (C), lateral raise (D), seated row (E), shoulder pulldown (F) and cough (G) at the mid-sternum at 2-, 8- and 14- weeks post-operatively.


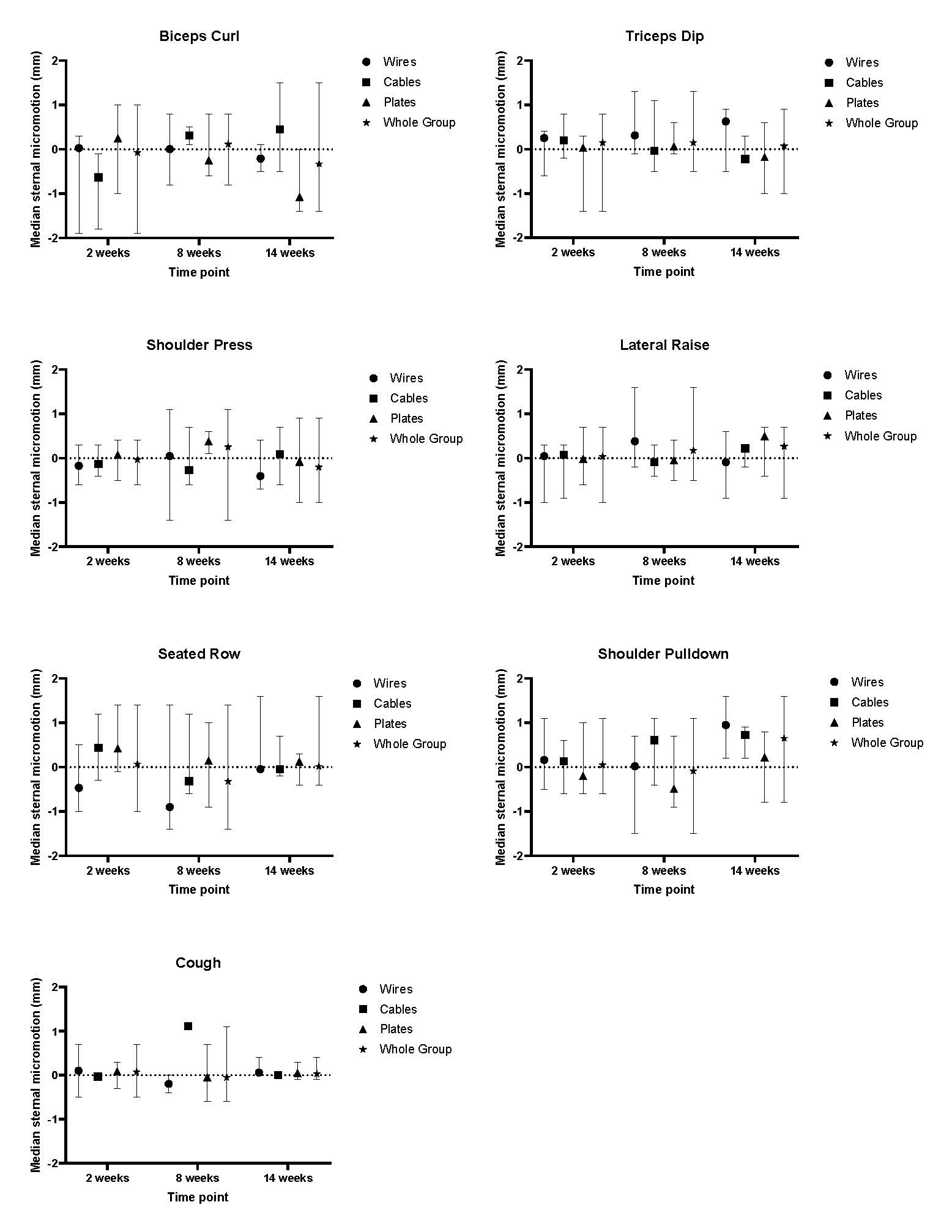


**A)**

**B)**

**C)**

**D)**

**E)**

**F)**

**G)**


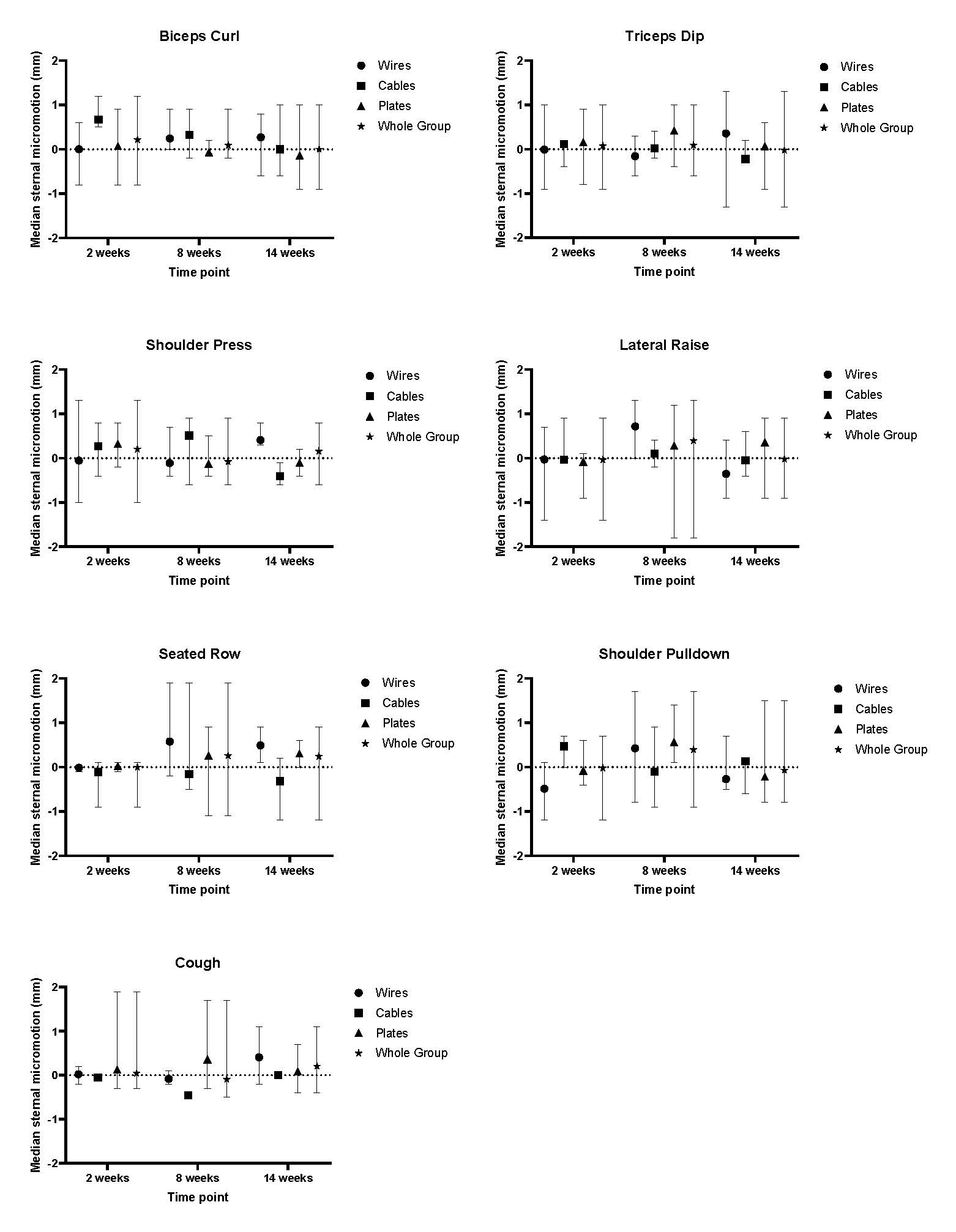


**A)**

**B)**

**C)**

**D)**

**E)**

**F)**

**G)**

**Supplementary Figure 4**. Changes in anterior-posterior motion at the sternal edges (mm) according to sternal closure mechanism (wires, cables or plates) during a biceps curl (A), triceps dip (B), shoulder press (C), lateral raise (D), seated row (E), shoulder pulldown (F) and cough (G) at the lower-sternum at 2-, 8- and 14- weeks post-operatively.
